# Patterns of hypothalamic regionalization in amphibians and reptiles: common traits revealed by a genoarchitectonic approach

**DOI:** 10.3389/fnana.2015.00003

**Published:** 2015-02-03

**Authors:** Laura Domínguez, Agustín González, Nerea Moreno

**Affiliations:** Faculty of Biology, Department of Cell Biology, University Complutense of MadridMadrid, Spain

**Keywords:** hypothalamus, prosencephalon, forebrain patterning, development, evolution

## Abstract

Most studies in mammals and birds have demonstrated common patterns of hypothalamic development highlighted by the combination of developmental regulatory genes (genoarchitecture), supporting the notion of the hypothalamus as a component of the secondary prosencephalon, topologically rostral to the diencephalon. In our comparative analysis we have summarized the data on the expression patterns of different transcription factors and neuroactive substances, used as anatomical markers, in the developing hypothalamus of the amphibian *Xenopus laevis* and the juvenile turtle *Pseudemys scripta*. This analysis served to highlight the organization of the hypothalamus in the anamniote/amniotic transition. We have identified supraoptoparaventricular and the suprachiasmatic regions (SCs) in the alar part of the hypothalamus, and tuberal and mammillary regions in the basal hypothalamus. Shared features in the two species are: (1) The supraoptoparaventricular region (SPV) is defined by the expression of Otp and the lack of Nkx2.1/Isl1. It is subdivided into rostral, rich in Otp and Nkx2.2, and caudal, only Otp-positive, portions. (2) The suprachiasmatic area contains catecholaminergic cell groups and lacks Otp, and can be further divided into rostral (rich in Nkx2.1 and Nkx2.2) and a caudal (rich in Isl1 and devoid of Nkx2.1) portions. (3) Expression of Nkx2.1 and Isl1 define the tuberal hypothalamus and only the rostral portion expresses Otp. (4) Its caudal boundary is evident by the lack of Isl1 in the adjacent mammillary region, which expresses Nkx2.1 and Otp. Differences in the anamnio-amniote transition were noted since in the turtle, like in other amniotes, the boundary between the alar hypothalamus and the telencephalic preoptic area shows distinct Nkx2.2 and Otp expressions but not in the amphibian (anamniote), and the alar SPV is defined by the expression of Otp/Pax6, whereas in *Xenopus* only Otp is expressed.

## The hypothalamus within the current prosomeric model

The hypothalamus is considered the forebrain territory par excellence dedicated to control homeostatic processes, and its neuroanatomical regionalization has been a much debated topic in recent years. The term “hypothalamus” was coined during the last century with the beginning of neuroanatomical studies (His, 1893a,b), following a columnar conception of the brain (Herrick, 1910; Kuhlenbeck, 1973). This was based on the idea that the forebrain is organized in longitudinal functional units homologous to the ones in the brainstem and it was considered that the ventricular sulci marked the straight longitudinal axis of the forebrain, ending somewhere in the telencephalon (Herrick, 1948; Kuhlenbeck, 1973). Following this concept and the analysis of classical “transverse” sections, the hypothalamus was defined as a diencephalic region beneath the thalamus (from the old Greek ÿpó: under). However, the hypothalamus is formed, as the rest of the forebrain, from the anterior neural plate through complex processes of morphogenesis. As a result, this brain region in the mature brain is highly distorted, mainly by the sharp flexure of the longitudinal brain axis and by differential degree of development of its components. These phenomena make it difficult to identify the basic units or subdivisions in the mature hypothalamus and understand the topological relationships between them. Moreover, the variable degree of elaboration and differentiation of structures in the hypothalamus of the different vertebrates obscures the interpretation of anatomical data and the comparison across species, and greatly complicates studies of forebrain evolution (Butler and Hodos, 2005; Bruce, 2008; Hodos, 2008; Nieuwenhuys et al., 2008).

Twenty years ago, the first proposal of the prosomeric model pointed out several evidences to discard the columnar paradigm of the forebrain organization, revealing the discrepancy between the traditional anatomical landmarks and the morphogenetic organization of the brain, what eventually led to refute the boundary role of the ventricular sulci (Puelles and Rubenstein, 1993; Rubenstein et al., 1994; Puelles, 1995). In this model, the forebrain is organized into transverse segments (prosomeres) and longitudinal zones defined by differential expression patterns of several developmental regulatory genes that establish the internal boundaries. According to the original prosomeric model and its subsequent revisions (Puelles and Rubenstein, 1993, 2003; Puelles, 1995, 2001; Puelles et al., 2012a) the hypothalamus is excluded from the diencephalon, which is composed of three neuromeres (prosomeres P1–P3). The rostralmost forebrain is designated the secondary prosencephalon that contains the hypothalamus (rostral to the diencephalic P3), the telencephalon impar, and the telencephalic hemispheres (Puelles and Rubenstein, 2003). The interpretation of the parts of the secondary prosencephalon is fraught with difficulties, mainly derived from the early optic and hemispheric evaginations and the different degree of development shown across vertebrates that disturb the primary pattern of this region (Nieuwenhuys et al., 2008). However, morphological, molecular, and hodological results have progressively contributed to highlight the organization of the main parts of the secondary prosencephalon and its subdivisions, particularly in mice, where different organization models have been proposed (Figdor and Stern, 1993; Puelles and Rubenstein, 2003; Shimogori et al., 2010; Diez-Roux et al., 2011; Morales-Delgado et al., 2011, 2014; Puelles et al., 2012a). Recently, in our group we applied similar developmental gene expression criteria to the identification of hypothalamic components in amphibians and reptiles (Moreno et al., 2012; Domínguez et al., 2013, 2014). We selected representative species of these vertebrate classes for their importance in evo-devo studies with a phylogenetic perspective. Amphibians constitute the only group of tetrapod anamniotes and represent a key model in anamniote/amniote transition, as they share features with other tetrapods (amniotes) and also with other anamniotes. In turn, reptiles occupy a crucial position, especially turtles, which were reported to be the most closely related to the extinct therapsids from which mammals arose (Northcutt, 1970), although, alternatively, they have been considered the sister group to crocodiles and birds (Zardoya and Meyer, 2001a,b). Therefore, the study of these vertebrate groups appears particularly relevant since the colonization of land by tetrapod ancestors is presumably one of the evolutionary events that could involve more neural changes.

Within the current anatomical context, we now define the hypothalamic boundaries with its neighboring forebrain areas on the basis of distinct molecular profiles during development. Thus, gene expression data have highlighted that the preoptic area does not belong to the hypothalamus but it is part of the subpallial telencephalic territory (Flames et al., 2007; Medina, 2008; Garcia-Lopez et al., 2009; Sánchez-Arrones et al., 2009; Zhao et al., 2009; Roth et al., 2010) and is topologically adjacent to the dorsal hypothalamic territory. The caudal hypothalamo-diencephalic boundary is highlighted by the distinct Six3, Lhx9, Arx and Dlx expression in the prethalamic territory (P3), as well as the Otx2 expression in the diencephalon, but not in the hypothalamus (Puelles et al., 2012a,b).

**Figure 1:**
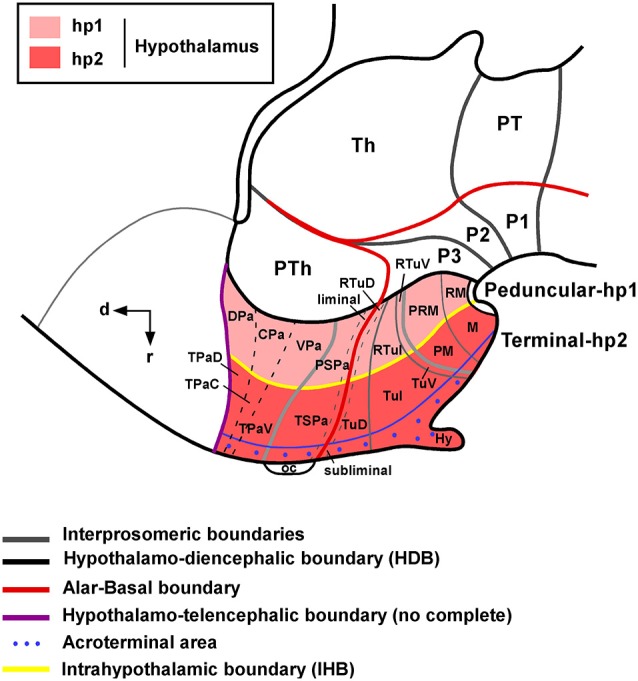
**Schematic representation of the mammalian hypothalamic organization according to the prosomeric model**. Note that the coordinate system for the hypothalamus rotates 90° because the longitudinal axis of the brain bends in the diencephalon. For abbreviations, see list. (Modified from Puelles et al., 2012a and Morales-Delgado et al., 2014).

The longitudinal domains of the alar and basal plates, which extend along the neuraxis, also extend to the hypothalamus and the alar–basal boundary is considered to end rostrally just behind the optic chiasm in all vertebrates (Puelles, 1995). The expression of the gene Nkx2.2 along the alar–basal boundary in the caudal prosencephalon continues rostrally in the hypothalamus, which allows distinguishing between alar and basal territories (Shimamura et al., 1995). The recently updated prosomeric model in mammals (see Figure 1) holds that the hypothalamus is subdivided dorsoventrally into alar, basal, and floor longitudinal domains and separates rostrocaudally, by the intrahypothalamic boundary (IHB), into two transverse regions called terminal hypothalamus (THy; rostral; hp2: hypothalamic prosomeric domain 2) and peduncular hypothalamus (PHy; caudal, hp1:hypothalamic prosomeric domain 1). The main forebrain bundles course dorsoventrally along PHy, which is also characterized by the generation of highly characteristic structures such as the main paraventricular nucleus, the retromammillary area and the migrated subthalamic nucleus (Puelles et al., 2012a). The THy contains the main tuberal and mammillary regions, as well as the supraoptic, suprachiasmatic, and retrochiasmatic areas. The THy includes a rostromedian subdomain recently named acroterminal area, with specializations such as the lamina terminalis (and related vascular organ), suprachiasmatic, and chiasmatic alar areas, and the anterobasal, arcuate, median eminence, and infundibular/neurohypophysial basal areas (Puelles et al., 2012a). During development, Six6 and Foxb1 gene expression apparently delineates the entire acroterminal territory. Although the structures included in the acroterminal part are obviously present in reptiles and amphibians (see ten Donkelaar, 1998a,b), developmental studies did not reveal specific markers for the origin of this hypothalamic part (Moreno et al., 2012; Domínguez et al., 2013, 2014).

In the alar hypothalamus, the dorsal domain is adjacent to the telencephalic preoptic area and expresses the transcrition factors Tbr1, Sim1 and Otp (Medina, 2008; Puelles et al., 2012a; Morales-Delgado et al., 2014). This subdomain is subdivided into terminal (and acroterminal) and peduncular areas, and among others, produces the paraventricular, supraoptic and periventricular nuclei. The second alar subdomain is located ventrally and is mainly characterized by the expression of Dlx genes. It is also subvivided into terminal (and acroterminal), and peduncular areas, which give rise to the suprachiasmatic nucleus, the anterior hypothalamic nucleus and the subparaventricular zone (Puelles et al., 2012a). The basal hypothalamus classically includes the tuberal (Tub) and mammillary regions (M), both mainly characterized by the expression of the morphogen Shh and the transcription factor Nkx2.1, with minor exceptions (Puelles et al., 2012a). The tuberal region (Tub) is divided into a proper tuberal area (terminal and acroterminal) and a retrotuberal area (peduncular), which produce the anterobasal and posterobasal nuclei in the dorsal portion, the ventromedial, dorsomedial and the arcuate nuclei in the intermediate portion and ventral portions. In the mammillary region, in addition to the proper mammillary and retromamillary regions (in the terminal and peduncular segmentes, respectively) a topologically dorsal band has been defined (called with the prefix peri-) that also included terminal and peduncular portions (see Figure 1).

## Hypothalamic organization in the anamnio-amniotic transition: evolutionary traits on hypothalamic regionalization

The achievement of new tools in developmental neuroanatomy for the analysis of the genoarchitecture of particular brain regions has led to the precise interpretation of the hypothalamic regionalization, and the definition of different hypothalamic progenitor domains, which were traditionally linked to anatomical landmarks that not always coincided with the molecular boundaries. Thus, the analysis of the patterns of distribution of main regulatory transcription factors and proteins involved in neural patterning, and that are also expressed after development, have allowed to determine the extent of the different hypothalamic histogenetic divisions. In addition, the molecular boundaries with the adjacent non-hypothalamic territories could be assessed. Comparative studies using the same sets of markers in different vertebrates, particularly amphibians and reptiles, have highlighted that the main molecular features of the subdivisions topologically identified in the hypothalamus have been highly conserved (Medina, 2008; Domínguez et al., 2010, 2011, 2013, 2014; Moreno and González, 2011; Morales-Delgado et al., 2011, 2014; Moreno et al., 2012). In the following sections the molecular characteristics of each of the main hypothalamic regions, as well as the boundaries with the neighboring areas are detailed for *Xenopus* and *Pseudemys* and will be compared with the situation found in other vertebrates (see Table 1).

**Table 1:** **Comparison of the different gene expression patterns detected in the different group of vertebrates**.

	Lamprey	Zebrafish	Lungfish	Xenopus	Turtle	Chick	Mouse
**POH**	——	——	——	——	Nkx2.2 (Moreno et al., 2012)	DIx5/Nkx2.2 (Bardet et al., 2006, 2010)	DIx2/Nkx2.2 (Flames et al., 2007)
**SPV**	-Pax6 (Murakami et al., 2001)		-Pax6 (Moreno and González, 2011)	-Pax6 (Moreno et al., 2008a; Domínguez et al., 2013)	Pax6 (Moreno et al., 2012)		Pax6 (Flames et al., 2007)
				Nkx2.2 (Domínguez et al., 2011, 2013)	Nkx2.2 (Moreno et al., 2012)		Nkx2.2 (Caqueret et al., 2006; Puelles et al., 2012a)
		Otp (Del Giacco et al., 2006; Blechman et al., 2007; Machluf et al., 2011; Herget et al., 2014)	Otp (Moreno and González, 2011)	Otp (Bardet et al., 2008; Domínguez et al., 2013)	Otp (Moreno et al., 2012)	Otp (Bardet et al., 2008)	Otp (Puelles and Rubenstein, 2003)
				Lhx5 (Domínguez et al., 2013)		Lhx5 (Abellán et al., 2010)	Lhx5 (Abellán et al., 2010)
**SC**		Nkx2.1/Shh (Rohr et al., 2001)	Nkx2.1 (Moreno and González, 2011)	Nkx2.1/2.2/Shh (Domínguez et al., 2010, 2011, 2013)	Nkx2.1/2.2 (Moreno et al., 2012)	Nkx2.1/2.2/Shh (Bardet et al., 2010)	-Nkx2.1/Shh (Puelles and Rubenstein, 2003; Medina, 2008)
	DIx (Martínez-de-la-Torre et al., 2011)	DIx (Medina, 2008)	IsI1 (Moreno and González, 2011)	Dlx/Isl1 (Brox et al., 2003; Moreno et al., 2008b; Domínguez et al., 2013)	Isl1 (Moreno et al., 2012)	Dlx/Isl1 (Abellán and Medina, 2009; Bardet et al., 2010)	DIx (Puelles and Rubenstein, 2003)
				Lhx1/Lhx7 (Moreno et al., 2004; Domínguez et al., 2013)		Lhx7 (Abellán and Medina, 2009)	Lhx1/Lhx7 (Abellán et al., 2010; Shimogori et al., 2010)
			Otp (Moreno and González, 2011)	Otp (Domínguez et al., 2014)	Otp (Moreno et al., 2012)	Otp (Bardet et al., 2008)	Otp (Morales-Delgado et al., 2011)
**Tub** **|** **|** **|**	Nkx2.1/Shh (Osorio et al., 2005; Medina, 2008)	Nkx2.1/Shh (Wolf and Ryu, 2013)	Nkx2.1/2.2 (Moreno and González, 2011)	Nkx2.1/2.2/Shh (Domínguez et al., 2010, 2011, 2014)	Nkx2.1 (Moreno et al., 2012) -Nkx2.2 (Moreno et al., 2012)	Nkx2.1/Shh (Medina, 2008; Abellán and Medina, 2009; Bardet et al., 2010)	Nkx2.1/2.2/Shh (Puelles and Rubenstein, 2003; Kurrasch et al., 2007; Medina, 2008; Morales-Delgado et al., 2011)
**BH** **|** **|** **|**	DIx (Martínez-de-la-Torre et al., 2011)		Isl1 (Moreno and González, 2011)	Dlx/Isl1 (Brox et al., 2003; Moreno et al., 2008b; Domínguez et al., 2014)	Isl1 (Moreno et al., 2012)		Dlx/Isl1 (Puelles and Rubenstein, 2003; Davis et al., 2004; Medina, 2008)
**Ma**					Lhx (Domínguez et al., 2014)			
	Nkx2.1 (Medina, 2008)	Nkx2.1 (Wolf and Ryu, 2013)	Nkx2.1 (Moreno and González, 2011)	Nkx2.1/2.2 (Domínguez et al., 2014)	Nkx2.1/2.2 (Moreno et al., 2012)	Nkx2.1 (García-Calero et al., 2008)	Nkx2.1 (Medina, 2008; Morales-Delgado et al., 2011)
		Otp (Wolf and Ryu, 2013)	Otp (Moreno and González, 2011)	Otp (Bardet et al., 2008; Domínguez et al., 2014)	Otp (Moreno et al., 2012)	Otp (Bardet et al., 2008)	Otp (Bardet et al., 2008)
				Shh (Domínguez et al., 2014)		Shh (García-Calero et al., 2008)	Shh (Morales-Delgado et al., 2011)
	-DIx (Martínez-de-la-Torre et al., 2011)			DIx (Domínguez et al., 2014)			-DIx (Puelles and Rubenstein, 2003; Medina, 2008)
	Lhx1/5 (Osorio et al., 2005)			Lhx1 (Domínguez et al., 2014)			Lhx1 (Shimogori et al., 2010)

*Note that we only have indicated negative expressions to remark a difference between groups of vertebrates that is not due to the lack of data in the literature (empty squares)*.

### Preoptohypothalamic boundary (POH)

**Figure 2:**
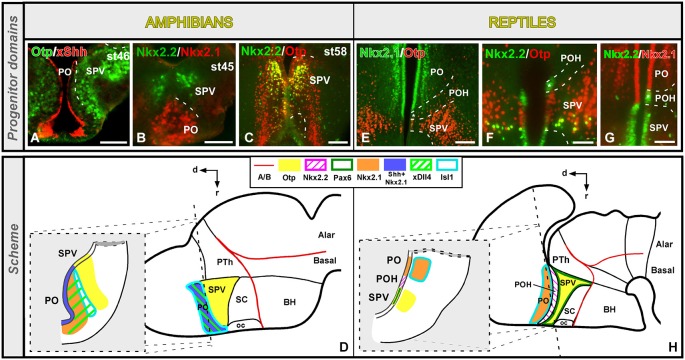
**Comparative aspects of the preoptic-hypothalamic (POH) boundary between amphibians and reptiles**. Photomicrographs of transverse sections through the developing preoptic-hypothalamic territory of *Xenopus* **(A–C)** and *Pseudemys* **(E–G)** illustrating its molecular profile based on the combinatorial expression of different transcription factors and neuropeptides indicated in each photomicrograph. The developmental stage in the cases of *Xenopus* is also marked. **(D)** and **(H)** are summarizing schemes of lateral views of the brains in which the main molecular features of the POH are illustrated according to the color code indicated. In both schemes, a transverse section through the level indicated on the lateral view of the brain is illustrated. Note that the coordinate system for the hypothalamus rotates 90° because the longitudinal axis of the brain bends in the diencephalon, and this is also the case for all photomicrographs of sagittal sections in all figures. For abbreviations, see list. Scale bars = 50 μm **(A,B)**, 100 μm **(C,F,G)**, 200 μm **(E)**.

The preoptic region (PO) was traditionally included within the hypothalamus until genoarchitectonic studies revealed that this region is derived from the FoxG1-positive telencephalic neuroephitelium (Tao and Lai, 1992; Murphy et al., 1994; Bourguignon et al., 1998; Zhao et al., 2009; Roth et al., 2010), revealing its subpallial nature (Flames et al., 2007; Medina, 2008; Garcia-Lopez et al., 2009; Moreno and González, 2011). In *Xenopus*, the PO is a Dll4, Isl1, Shh and Nkx2.1 positive territory that limits ventrally with the Otp expressing supraoptoparaventricular region (SPV, the dorsal part of the alar hypothalamus; Domínguez et al., 2013). This gene expression profile of the subpallial PO seems to be largely shared by reptiles (*Pseudemys*: Moreno et al., 2010) and the rest of amniotes (Puelles et al., 2000). However, some differences can be noted regarding its ventral boundary with the hypothalamus. In *Xenopus*, the Nkx2.1/Shh positive PO is in contact with the Otp/Nkx2.2 positive SPV (Figures 2A–D; Domínguez et al., 2013). In contrast, a narrow Nkx2.2 positive territory has been observed in the turtle (and not in *Xenopus*) between the Isl1/Nkx2.1 positive PO and the SPV-Otp expressing region (Figures 2E–H; Moreno et al., 2012). In this context, recent studies have described a Dlx/Nkx2.2 expressing band in mammals and birds that represents the boundary between the PO and the hypothalamus (Bardet et al., 2006, 2010; Flames et al., 2007), like in the turtle but in contrast to the situation found in amphibians and fishes (Domínguez et al., 2011, 2013; Moreno et al., 2012). Thus, the presence of this Nkx2.2 positive territory (preopto-hypothalamic boundary) supposes a relevant acquisition during the anamnio-amniotic transition.

### Supraoptoparaventricular region (SPV)

The SPV is the most dorsal region in the alar hypothalamus and it is defined by the expression of Otp/Sim1 and the lack of Dlx/Shh/Nkx2.1 expression in all vertebrates analyzed (reviewed in Markakis, 2002; Medina, 2008; Moreno and González, 2011; Puelles et al., 2012a), from anamniotes (Brox et al., 2003; Del Giacco et al., 2006; Blechman et al., 2007; Bardet et al., 2008; Domínguez et al., 2010, 2013; Machluf et al., 2011; Martínez-de-la-Torre et al., 2011; Herget et al., 2014) to amniotes (Acampora et al., 1999; Flames et al., 2007; Bardet et al., 2008; Morales-Delgado et al., 2011, 2014).

**Figure 3:**
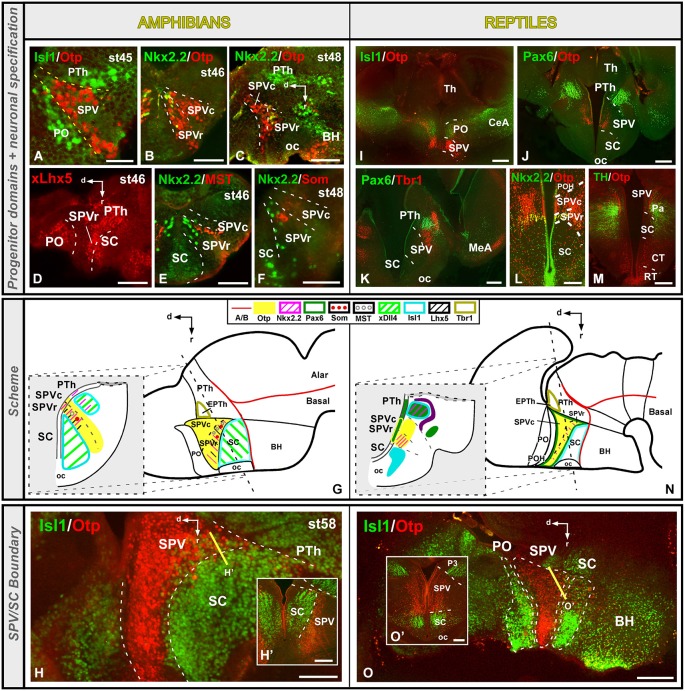
**Comparative aspects of the supraoptoparaventricular (SPV) region between amphibians and reptiles**. Photomicrographs of transverse **(A,B,E,F,H’,I–M,O’)** and sagittal **(C,D,H,O)** sections through the developing SPV territory of *Xenopus* **(A–H’)** and *Pseudemys* **(I–O’)** illustrating its molecular profile based on the combinatorial expression of different transcription factors and neuropeptides indicated in each figure. The developmental stage in the cases of *Xenopus* is also marked. **(G)** and **(N)** are summarizing schemes of lateral views of the brains in which the main molecular features of the SPV are illustrated according to the color code indicated. In both schemes, a transverse section through the level indicated on the lateral view of the brain is illustrated. Scale bars = 50 μm **(A–F)**, 100 μm **(H’)**, 200 μm **(H,L,O,O’)**, 500 μm **(I–K,M)**.

Both in the amphibian *Xenopus laevis* and in the turtle *Pseudemys scripta* the extent of the SPV is particularly well defined by the expression of the transcription factor Otp, and its dorsal limit with the PO is defined by the lack of Isl1 expression (Figures 3A,G,I,N; Bardet et al., 2008; Moreno et al., 2012; Domínguez et al., 2013). The boundaries of the Otp positive SPV with the adjacent prethalamic and SC territories are also discernible by the lack of Isl1 in the SPV (Figures 3G,H,H’,N,O,O’; Moreno et al., 2012; Domínguez et al., 2013). The caudal boundary with the Tbr1-expressing prethalamic eminence (EPTh in P3) is also extremely conserved in the anamnio-amniotic transition (Figures 3G,N; Moreno et al., 2012; Domínguez et al., 2013). Thus, the lack of Dlx, Shh, Isl1, and Nkx2.1 in the SPV of all the vertebrates analyzed is a constant feature in tetrapods that allows the distinction of the SPV territory from the adjacent diencephalic prethalamus (PTh) and SC area (Flames et al., 2007; Medina, 2008; van den Akker et al., 2008; Domínguez et al., 2010, 2013; Moreno and González, 2011; Moreno et al., 2012; Puelles et al., 2012a; Morales-Delgado et al., 2014).

Interestingly, in some groups of fishes such as teleosts and lungfishes (the closest living relatives of tetrapods) the expression of Otp in the SPV territory has been reported (Del Giacco et al., 2006; Blechman et al., 2007; Machluf et al., 2011; Moreno and González, 2011). Moreover, a very recent study of the molecular and neurochemical features of this hypothalamic region in zebrafish identified a territory homologous to the mammalian paraventricular nucleus called neurosecretory preoptic-hypothalamic area (NPO), which is characterized by the expression of Otp and the lack of Isl1, Dlx5 and Arx, which define its anatomical boundaries (Herget et al., 2014).

Furthermore, both in *Xenopus* and *Pseudemys* the expression of the transcription factor Nkx2.2, allowed the rostro-caudal subdivision of the SPV into two different progenitor domains (Domínguez et al., 2011, 2013; Moreno et al., 2012). The rostral domain (SPVr) is characterized by the ventricular and subventricular expression of Otp and Nkx2.2, whereas the caudal domain (SPVc) only expresses Otp (Figures 3B,C,G,L,N; Moreno et al., 2012; Domínguez et al., 2013). In the case of *Xenopus*, Lhx5 has also been distinctly observed in the rostral domain of the SPV (Figure 3D; Domínguez et al., 2013), in agreement with descriptions of the SPV of mouse and chick (Bulfone et al., 1993; Abellán et al., 2010). Actually, attending to its internal molecular organization, this region in mammals and birds was divided rostrocaudally into terminal and peduncular portions (Bardet et al., 2008; Puelles et al., 2012a). In addition, regarding to the localization of the Nkx2.2, positive cells, they were reported partially overlapping the Sim1 expression domain in the anterior hypothalamus of the mouse, where the Nkx2.2 cell population seems to occupy a rostral position (see Figures 5K, 7D,E in Caqueret et al., 2006). And a population of Nkx2.2, expressing cells located in the ventral part of the paraventricular area in mouse has been described (Puelles et al., 2012a). In mammals, it was described that Nkx2.2 cells from the dorsal portion of the peduncular tuberal hypothalamus migrated very early colonizing this paraventricular nucleus, overlapping the Otp expressing cells (Puelles et al., 2012a).

The SPV of mammals and birds is also characterized by the expression of Pax6 and Tbr1 (Michaud et al., 1998, 2000; Puelles and Rubenstein, 2003; Flames et al., 2007; Medina, 2008). In the turtle, Pax6 expression was demonstrated in the ventricular zone of the SPV region (Figures 3J,K,N; Moreno et al., 2012), whereas it was not observed in *Xenopus* (Moreno et al., 2008a, 2012; Domínguez et al., 2013).The lack of Pax6 expression in the SPV has also been reported in other anamniotes (Murakami et al., 2001; Moreno and González, 2011). This situation might reflect differences in the specification of this area between amniotes and anamniotes. Therefore, the expression of Pax6 seems to have appeared for the first time in amniotes, and it could be related to the specific size and functionality of this area, being Pax6 involved in the dorsoventral brain organization, as it has been demonstrated in the mammalian forebrain (Toresson et al., 2000).

Of interest, some studies have recently described in amniotes a contingent of Otp positive cells generated in the SPV that migrate into the medial amygdala (Bardet et al., 2008; Abellán et al., 2010; García-Moreno et al., 2010; Bupesh et al., 2011a,b; Medina et al., 2011). This most likely represents a conserved feature that arose early in phylogeny since in lungfishes, anurans, and reptiles Otp expressing cells have been consistently observed in the region identified as the medial amygdala (González and Northcutt, 2009; Moreno et al., 2010; Domínguez et al., 2013), and a similar migratory pathway from the SPV was suggested (Moreno and González, 2011).

Regarding its neurochemical profile, the SPV of amphibians and reptiles contains different groups of cells secreting several neuropeptides such as vasotocine, mesotocine CRH, and TRH (Smeets et al., 1990; Propper et al., 1992; D’Aniello et al., 1999; Domínguez et al., 2008; López et al., 2008) that constitute part of the neuroendocrine hypothalamic system, which seems to be very conserved during the anamnio-amniotic transition. The TRH positive population in *Xenopus* and turtle, is specifically located within the Otp expressing territory (Domínguez et al., 2008; López et al., 2008), suggesting that this transcription factor might be involved in the specification of the TRH phenotype during the anamnio-amniotic transition, as describing in other amniotes (Goshu et al., 2004; Del Giacco et al., 2008; Morales-Delgado et al., 2014). Moreover, in *Xenopus* a correlation between the emergence of somatostatin and mesotocine positive neurons and the presence of Otp was observed in the SPV (Figures 3E,F; Domínguez et al., 2013), highlighting the role of this transcription factor in the specification of these postmitotic cell populations and, consequently, in the differentiation of independent nuclei within this territory. In mice it was shown that Otp is involved in the specification of the somatostatin expressing cell populations (Morales-Delgado et al., 2011) and, therefore, the specification of the somatostatin phenotype in the SPV seems to be conserved during the vertebrtae evolution. Of note, in fishes Otp might also be involved in the specification of multiple neurosecretory hypothalamic cell populations such as those containing somatostatin, vasoticin-neurophysin and isotocin-neurophysin (the latter two are homologous of mammalian vasopressin and oxytocin, respectively), as suggested by the overlapping of these neurosecretory populations in the Otp expressing domain (Blechman et al., 2007; Eaton and Glasgow, 2007; Tessmar-Raible et al., 2007; Eaton et al., 2008; Herget et al., 2014). Finally, a population of dopaminergic cells was described in the paraventricular nucleus of the SPV in reptiles (Figure 3M; Smeets et al., 1987), whereas catecholaminergic cells (TH positive) were not found in the SPV of *Xenopus* (Domínguez et al., 2013).

### Suprachiasmatic region (SC)

**Figure 4:**
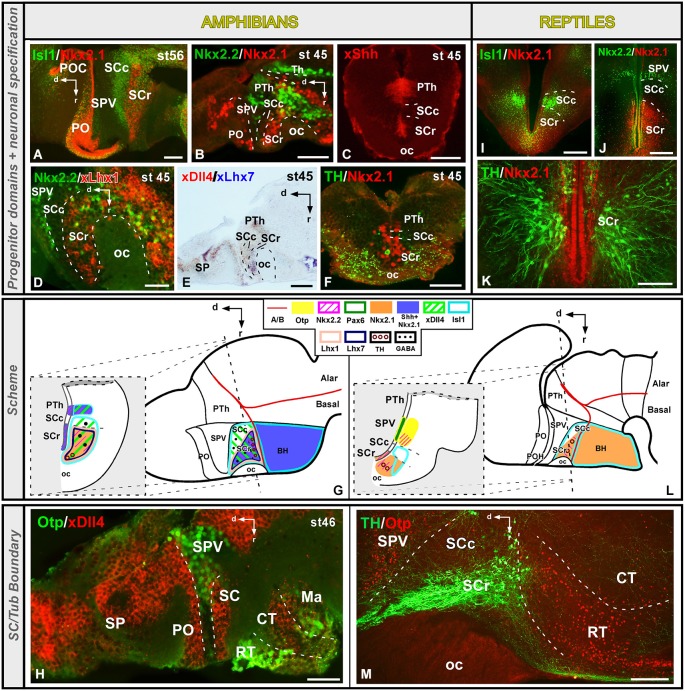
**Comparative aspects of the suprachiasmatic (SC) territory between amphibians and reptiles**. Photomicrographs of transverse **(C,F,I–K)** and sagittal **(A,B,D,E,H,M)** sections through the developing SC territory of *Xenopus* **(A–H)** and *Pseudemys* **(I–M)** illustrating its molecular profile based on the combinatorial expression of different transcription factors and neuropeptides indicated in each figure. The developmental stage in the cases of *Xenopus* is also marked. **(G)** and **(L)** are summarizing schemes of lateral views of the brains in which the main molecular features of the SC region are illustrated according to the color code indicated. In both schemes, a transverse section through the level indicated on the lateral view of the brain is illustrated. Scale bars = 25 μm **(D,H)**, 50 μm **(B,C,E,F)**, 100 μm **(A,K)**, 200 μm **(I,J,M)**.

The SC constitutes the ventral part of the alar hypothalamus and contains important neuroendocrine cell groups. This region is characterized by the expression of Dlx/Arx genes in all vertebrates analyzed (Bachy et al., 2002; Brox et al., 2003; Puelles and Rubenstein, 2003; Flames et al., 2007; Bardet et al., 2008, 2010; Medina, 2008; Domínguez et al., 2011, 2013; Martínez-de-la-Torre et al., 2011). The SC abuts ventrally the basal hypothalamus, characterized by the expression of Shh/Nkx2.1 genes, defining the alar–basal boundary, according to the prosomeric model (Puelles et al., 2012a). Both in amphibians and reptiles, this ventral boundary is highlighted by the Otp expression in the most rostral part of the Tub (Figures 4G,H,L,M; Moreno et al., 2012; Domínguez et al., 2013). Caudally, in *Xenopus*, the SC region is adjacent to the Dll4 positive PTh in the diencephalon, whereas in the juvenile turtle the SPV Otp-expressing cells extends reaching the alar–basal boundary (Figures 4G,L; Moreno et al., 2012; Domínguez et al., 2013).

This region in mammals, identified as subparaventrcular area (Puelles et al., 2012a), is defined as a Dlx+/Nkx2.1-territory (Puelles and Rubenstein, 2003; Medina, 2008; Puelles et al., 2012a). Furthermore, a recent study in mice has defined a territory called intrahypothalamic diagonal band, based primarily on the differential expression of Lhx1, Lhx6, Lhx7 and Lhx8, constituting the territory from which the suprachiasmatic populations of interneurons would arise (Shimogori et al., 2010). The domain recently defined in mammals as the liminal subparaventricular subdomain distinctively contains alar mantle cells expressing Nkx2.1, not present at the supraliminal subdomain (Shimogori et al., 2010; Puelles et al., 2012a). It could be comparable to those results showed in our models, but in our case the Nkx2.1 expression is also detected in the ventricular cells.

The acroterminal domain in front of the terminal region was proposed to be the source of the proper suprachiacmaitc nucleus (Puelles et al., 2012a). Both in *Xenopus* and *Pseudemys*, no distinct labeling paterns could help in the deliniation of the acroterminal part of the alar hypothalamus but the specialized derivatives of this region have been classically described, such as the optic chiasm, postchiasmatic commissures and suprachiasmatic nuclei (for review, see ten Donkelaar, 1998a,b). Therefore, it is likely that the origin in the terminal region would be conserved, but further studies are needed in order to identify genoarchitectonically this territory.

In birds, Nkx2.1 is also expressed in the subparaventricular nucleus, that belongs to the suprachiasmatic domain, which also expresses Nkx2.2, Lhx6/7 and Lhx8 (Abellán and Medina, 2009; Bardet et al., 2010). This region of *Xenopus* and turtle is Isl1-positive (Figures 4A,G,I,L; Moreno et al., 2008b, 2012; Domínguez et al., 2013) and, in the case of *Xenopus* in which the expression of several Dlx genes has been analyzed, the Isl1 and Dlx expression domains overlap in almost all prosencephalic regions including the entire SC territory (Brox et al., 2003; Domínguez et al., 2010, 2013). The transcription factors Nkx2.1 and Nkx2.2, are also expressed in the SC (Figures 4B,G,J,L; Domínguez et al., 2010, 2011) and the combination of both markers allowed the identification of rostro-caudal subdivisions in *Xenopus* and *Pseudemys*. Thus, only in the rostral part (SCr) Nkx2.1 and Nkx2.2 are found in the ventricular zone, in contrast to the caudal portion (SCc) that is devoid of expression (Figures 4B,J; Moreno et al., 2012; Domínguez et al., 2013). Of note, in *Xenopus*, the expression pattern of the morphogen Shh in SC runs parallel to the Nkx2.1 expressing domain (Figures 4C,G), suggesting a regulatory role of Shh through Nkx2.1 actions also in amphibians (Domínguez et al., 2013). Also in *Xenopus*, the expression of Lhx1 and Lhx7 is restricted to the rostral SC domain (Figures 4D,E,G; Moreno et al., 2004; Domínguez et al., 2013), which would be comparable to the subparaventricular nucleus described in chicken (Abellán and Medina, 2009; Bardet et al., 2010). In the case of fishes, expression of Dlx and Lhx7 has been detected in comparable regions to the SC territory in *Medaka* (Alunni et al., 2004). In addition, expression of Dlx genes in the SC primordium has been observed in the lamprey (Martínez-de-la-Torre et al., 2011), and Isl1 and Nkx2.1 expressions have been detected in the SC region of lungfishes (Moreno and González, 2011). However, the region expressing Shh/Nkx2.1 in fishes extends to the entire SC territory and subdivisions were not observed (Rohr et al., 2001).

Regarding the Nkx2.1 expression in the alar hypothalamus, it appears that is gradually restricted especially in the SC, from amphibians through amniotes (Figure 7). In mammals, the Lhx6 + intrahypothalamic diagonal band, proposed by Shimogori et al. (2010) corresponds to the Nkx2.1 expressing band of Puelles in the subparaventricular region of the alar domain (see Figure 8.9D in Puelles et al., 2012a). Also in mammals, like in Xenopus and turtle, this small band matches the Nkx2.2 expression (see Figure 8.9F in Puelles et al., 2012a). It could be related to the liminal subparaventricular area proposed by Puelles et al. (2012a). Thus, Nkx2.1 and Shh are expressed in almost the SC territory in non-tetrapod anamniotes like the zebrafish (Rohr et al., 2001), whereas in non-mammalian tetrapods like the anamniote *Xenopus* and the amniote reptiles and birds the Nkx2.1 expression is restricted to just a SC subdomain (Medina, 2008; van den Akker et al., 2008; Abellán and Medina, 2009; Moreno et al., 2012; Domínguez et al., 2013). Consequently, the evolutionary tendency of the disappearance of both developmental regulators in the SC begins at the origin of the tetrapod phylogeny. Furthermore, this progressive disappearance of Shh/Nkx2.1 expression in the alar hypothalamus/SC region has been related to the pallial expansion that takes place in amniotes (Bruce and Neary, 1995; Striedter, 1997), and the reduction of the alar hypothalamus in amniotes in contrast to anamniotes at the expense of the thalamic expansion (van den Akker et al., 2008; for review, see Medina, 2008).

**Figure 5:**
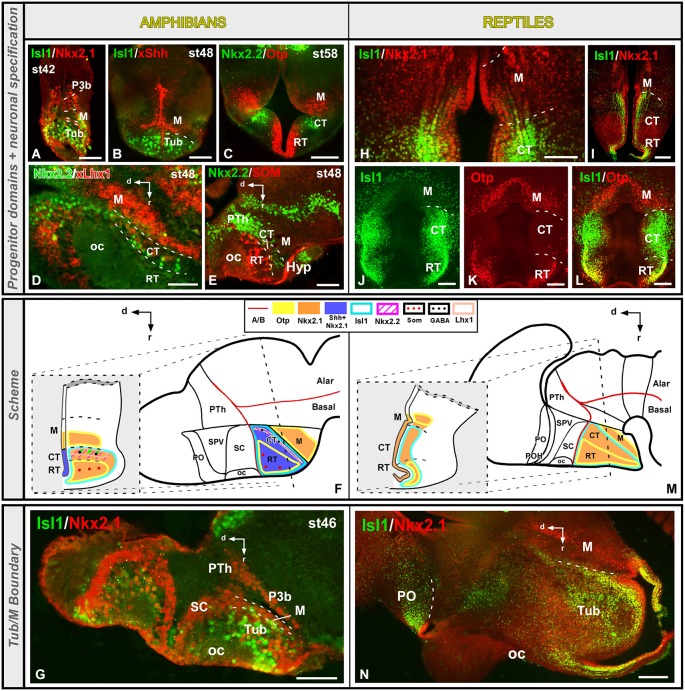
**Comparative aspects of the tuberal (Tub) territory between amphibians and reptiles**. Photomicrographs of transverse **(A–C,H–L)** and sagittal **(D,E,G,N)** sections through the developing Tub territory of *Xenopus* **(A–G)** and *Pseudemys* **(H–N)** illustrating its molecular profile based on the combinatorial expression of different transcription factors and neuropeptides indicated in each figure. The developmental stage in the cases of *Xenopus* is also marked. **(F)** and **(M)** are summarizing schemes of lateral views of the brains in which the main molecular features of the Tub region are illustrated according to the color code indicated. In both schemes, a transverse section through the level indicated on the lateral view of the brain is illustrated. Scale bars = 100 μm **(A–E,G,J–L,N)**, 200 μm **(H)**, 500 μm **(I)**.

**Figure 6:**
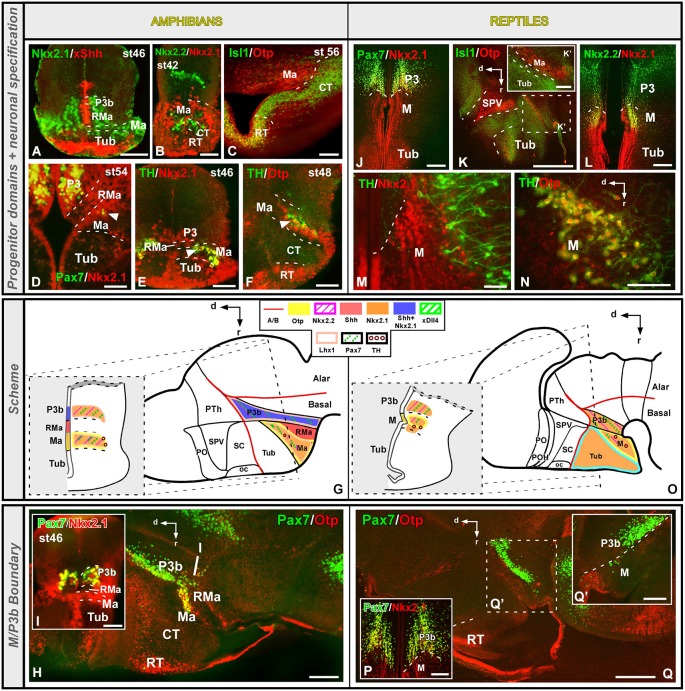
**Comparative aspects of the mammillary (M) territory between amphibians and reptiles**. Photomicrographs of transverse **(A–F,I,J,L,M,P)** and sagittal **(H,K,N,Q,Q’)** sections through the developing tuberal territory of *Xenopus* **(A–I)** and *Pseudemys* **(J–Q’)** illustrating its molecular profile based on the combinatorial expression of different transcription factors and neuropeptides indicated in each figure. The developmental stage in the cases of *Xenopus* is also marked. **(G)** and **(O)** are summarizing schemes of lateral views of the brains in which the main molecular features of the M region are illustrated according to the color code indicated. In both schemes, a transverse section through the level indicated on the lateral view of the brain is illustrated. Scale bars = Scale bars: 100 μm **(A–F,H,I,K’,M)**, 200 μm **(L,N,P,Q,Q’)**, 500 μm **(J,K)**.

**Figure 7:**
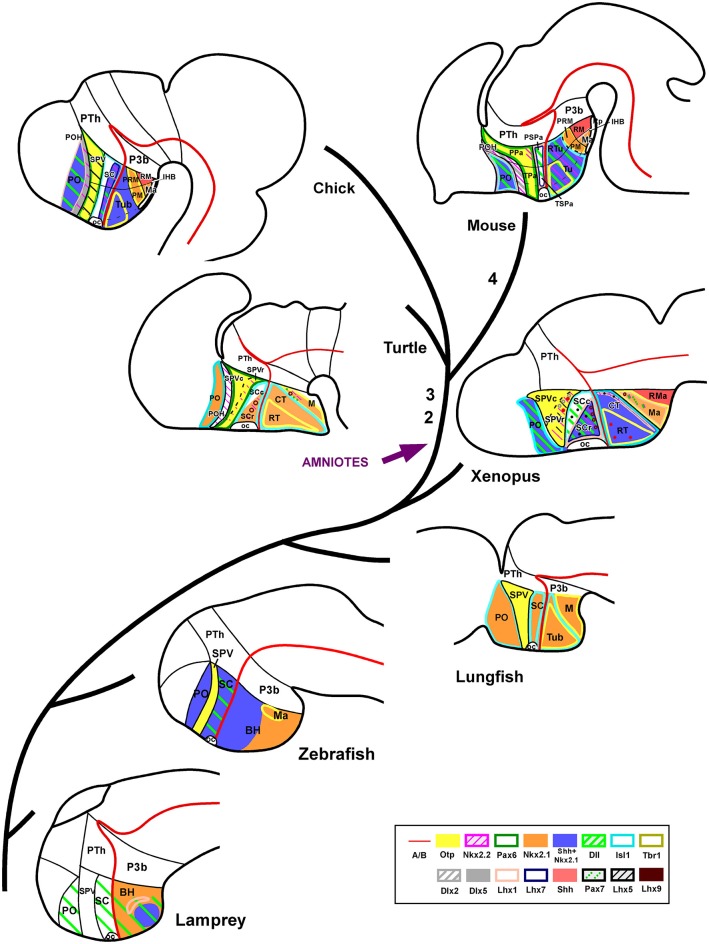
**Phylogenetic diagram representing the regionalization of the hypothalamus based on molecular criteria**. Representative species of different vertebrate groups are considered, including an agnathan fish (lamprey), a teleost fish (zebrafish), a dipnoi (lunghfish), an anuran amphibian (*Xenopus*), a reptile (turtle), a bird (chicken), and a mammal (mouse). In all species, the hypothalamus includes comparable molecular compartments, and each compartment shows a tendency to a common organization regarding its molecular expression profile. However, there are some remarkable differences in the expression patterns during evolution, such as the lack of Pax6 expression in the SPV of lamprey, lunghfish and *Xenopus*; the SC in mammals virtually does not express Shh/Nkx2.1 that are expressed in non mammalian amniotes and in anamniotes; Otp is expressed in the mammillary region of all vertebrates analyzed (no data in the lamprey are available). However, most differences in the scheme are due to the absence of data in the literature. The numbers 1–4 in the scheme represent the main evolutionary events regarding to the hypothalamic organization, as follows: (1) Nkx2.1 expression restriction in SC. (2) POH Nkx2.2 expression. (3) Pax6 expression in SPV for the first time. (4) Pallial and thalamic expansion at the expense of the alar hypothalamic reduction. Note that the developmental stages used in the scheme are not equivalent for all species.

In functional terms, the SC region is known to belong to the neuroendocrine system and therefore consists of multiple neuropeptide-secreting cell populations. In mammals and birds, the SC is characterized by the presence of TRH positive cells, among others, that have also been reported in anamniotes such as anurans and fishes (Domínguez et al., 2008), but that were not detected in reptiles (López et al., 2008). However, the SC region in amphibians and reptiles is characterized by the presence of catecholaminergic cell groups (González and Smeets, 1991; González et al., 1993) restricted to the rostral domain (Figures 4F,G,K,L; Morona and González, 2008; Moreno et al., 2012; Domínguez et al., 2013). This feature appears to be conserved throughout vertebrate evolution, being observed in amniotes and anamniotes (Hökfelt et al., 1984; González et al., 1993; Smeets and González, 2000; Moreno et al., 2012; Domínguez et al., 2013). In addition, the GABAergic expression has been analyzed in the SC of *Xenopus* showing that it is widely distributed along the entire Dlx expressing zone (Domínguez et al., 2013), suggesting that Dlx could be involved in the GABAergic specification, like in mammals (Price et al., 1991; Bulfone et al., 1993; Marín and Rubenstein, 2001).

### Tuberal region (Tub)

This region is currently considered to extend in the dorsal part of the basal hypothalamus, and like the rest of the hypothalamus has been postulated that posses acroterminal, terminal, and peduncular portions (Figure 1; Puelles et al., 2012a). The Tub is primarily characterized by the expression of Shh, which is directly involved in the organization of the basal hypothalamus through Nkx2.1 action (Kimura et al., 1996; Puelles et al., 2004), and thus Shh/Nkx2.1 expression has been observed in all the vertebrates analyzed (reviewed in Medina, 2008; Moreno and González, 2011). In amphibians and reptiles this territory is defined by the expression of Nkx2.1 and Isl1 (Figures 5A,B,F,H–J,M; Moreno et al., 2008b, 2012; Domínguez et al., 2014). Moreover, in *Xenopus* the Tub is also characterized by the ventricular expression of Shh, along with Nkx2.1 (Figures 5B,F; Domínguez et al., 2010, 2014), suggesting that also in *Xenopus* Shh could be implicated in the hypothalamic organization by the action of Nkx2.1, as in amniotes (Kimura et al., 1996; Puelles et al., 2004; reviewed in Medina, 2008). Consistently, both in *Xenopus* and *Pseudemys* the transcription factor Otp is exclusively located in the rostral tuberal portion (RT), within the Isl1-positive basal territory (Figures 5C,F,K–M).

However, in mammals the dorsal portion of the rostral terminal Tub is the only Otp expressing zone (Morales-Delgado et al., 2014), and the transcription factor Isl1 is expressed in the ventromedial and arcuate nuclei, where it is involved in the hypothalamic development and the regulation of the reproductive behavior (Davis et al., 2004). Moreover, the intermediate terminal and peduncular Tubs of mammals are also characterized by the expression of Dlx genes, involved in the specification of the GABAergic cell fate (Yee et al., 2009). Importantly, the transcription factor Otp is expressed in the dorsal Tub of mouse and in clusters of cells that from the acroterminal domain give rise to part of the nucleus arcuatus (Puelles et al., 2012a). Thus, the particular expression of Otp in a subdomain of the Tub appears as a conserved feature of the basal hypothalamus in amniotes and anamniotes equivalent to the region that gives rise to part of the arcuate nucleus in amniotes (Bardet et al., 2008; Puelles et al., 2012a).

Distinctly, the caudal tuberal part (CT) of *Xenopus* and *Pseudemys* is characterized by the lack of Otp expression (Figures 5C,F,K–M) and, expression of Nkx2.2 (Figures 5C–F), a transcription factor typically located in basal territories and necessary to maintain the ventral phenotype (Briscoe and Ericson, 1999; Sander et al., 2000; Garcia-Lopez et al., 2004; Puelles et al., 2004). Moreover, in mammals, Nkx2.2 in this Tub has been detected in the ventromedial nucleus and the core portion of the dorsomedial peduncular hypothalamic nucleus, in the terminal and peduncular domains respectively (Puelles et al., 2012a), where it is involved in the specification of the basal phenotype and the specification of the ventromedial fate (Kurrasch et al., 2007). Thus, it is possible that the Otp/Nkx2.1/Nkx2.2 expression in Xenopus likely resembles the mouse situation in which Otp is expressed in the dorsal domain of the terminal tuberal hypothalamus (likely including the acroterminal domain; Puelles et al., 2012a; Morales-Delgado et al., 2014), whereas Nkx2.1 is found in the terminal and peduncular intermediate portion (TuI), which gives rise to the ventromedial and dorsomedial nuclei (Puelles et al., 2012a), and likely corresponds to the region with Nkx2.2, Lhx1 and Dll4 expression found in our models, thus resembling the subdivision proposed (Figures 5D,F; Domínguez et al., 2014).

The boundary between the tuberal and mammillary territories in the basal hypothalamus of amphibians and reptiles is mainly defined by the lack of Isl1 expression in the mammillary region within the continuous Nkx2.1 positive tuberomammillar region (Figures 5F,G,M,N; Moreno et al., 2012; Domínguez et al., 2014). In addition, both regions can be distinguished by the differential expression of the transcription factor Otp, which is expressed in the mammillary region and not in the caudalmost tuberal domain (Figures 5C,F,L,M; Moreno et al., 2012; Domínguez et al., 2014). In *Pseudemys* a thin Nkx2.1 positive band (Moreno et al., 2012) can be detected between the Isl1 positive caudal tuberal zone and the Otp expressing mammillary region, defining specifically the tuberomammillary boundary (Moreno et al., 2012). In mammals, a thin ventral band expressing Dlx5/Nkx2.1/Arx+ but without Otp expression has been recently described in the ventral tuberal domain (Puelles et al., 2012a; Morales-Delgado et al., 2014). This is associated with a longitudinal circumventricular organ and it is the source of histamine in the hypothalamic cells (Puelles et al., 2012a).

Compared to tetrapods, there are only a few data about the hypothalamic organization in fishes, mainly attending to expression patterns and development. Recent studies in lunghfishes have revealed that Nkx2.1 and Isl1 are expressed in the entire Tub, whereas Otp expression is restricted to the most rostral and dorsal part, sustaining similar subdivisions in the tuberal territory to the ones described in tetrapods, using the same markers (Moreno and González, 2011). Data obtained in agnathans (lampreys) demonstrated that a substantial number of Dlx expressing cells occur in the tuberal hypothalamic nucleus and the tuberomammillary region, similar to anurans (Martínez-de-la-Torre et al., 2011).

The chemoarchitecture and neuronal specification processes in the Tub seem to be largely conserved throughout vertebrate evolution. In *Xenopus*, in the Otp-positive rostral Tub a population of somatostatin expressing cells has been observed (Figures 5E,F; Domínguez et al., 2014) suggesting the implication of Otp in the specification of this neurons in anurans, as has been previously reported in mammals (Acampora et al., 1999; Wang and Lufkin, 2000). In the mouse, the anterobasal nucleus, in the acroterminal domain defined by Puelles et al. (2012a), has been described to be the source of somatostatin cells to the ventromedial and arcuatus nucleus, where Otp would be specifically involved in the specification of these neurons (Morales-Delgado et al., 2011). The neuropeptide TRH, has been traditionally located in the dorsomedial nucleus (Hökfelt et al., 1975; Lechan et al., 1986; Tsuruo et al., 1987; Merchenthaler et al., 1988) and in the lateral hypothalamic area of mammals, where a recent study has proved its role in the arousal generation (Horjales-Araujo et al., 2014). However, a recent study has revealed that these tuberal TRH positive populations are likely generated in the SPV alar region (Morales-Delgado et al., 2014). The neuromodulator TRH has also been found in the periventricular hypothalamic nuclei of reptiles and in the Tub of anurans (Domínguez et al., 2008; López et al., 2008).

Finally, the anatomical position of the Dll4 expressing cell group located in the most caudal tuberal part of *Xenopus* is closely related to the GABAergic positive population (unpublished data), suggesting an implication of Dll4 in the specification of the GABAergic phenotype in the Tub, as occurs in the majority of the histogenetic domains where both markers colocalize (Price et al., 1991; Bulfone et al., 1993; Marín and Rubenstein, 2001). A distinct feature of the neurochemical profile of the Tub in birds is the presence of catecholaminergic populations originated under the control of Shh. Actually, in the chicken a dopaminergic positive population located in the Tub exists that is specified by Shh in a Six3-dependent manner (Ohyama et al., 2005). However, in anurans and reptiles there are not catecholaminergic cells in the Tub (Moreno et al., 2012; Domínguez et al., 2014).

### Mammillary region (M)

In the current prosomeric model, the mammillary region is interpreted as formed by mammillary-terminal and retromammillary-peduncular regions (see Figure 1; Puelles et al., 2012a). In addition, immediately dorsal to them, corresponding peri-mammillary and peri-retromammillary regions (RMas) were considered. The latter form in mouse a rostral (peri-) band, which has been described based on the Otp/Nkx2.1 expression and the lack of Dlx genes (Puelles et al., 2012a).

In recent years, the regionalization of the this area has been under analysis and the terminology used for its various subdivisions and their actual extent in the basal hypothalamic region have progressively varied with the appearance of the molecular approach (Shimogori et al., 2010; Puelles et al., 2012a). By means of the combinatorial expression of Shh and Nkx2.1, in *Xenopus* two different regions were identified, the mammillary area proper (Ma) that is Otp/Nkx2.1+/Shh-, and the RMa, where the expression patterns of Shh and Nkx2.1 are inverted, being Nkx2.1-/Shh+ (Figures 6A,G; Domínguez et al., 2014). In contrast to *Xenopus*, in the turtle the Nkx2.1 expression is continuous throughout the mammillary band, abutting directly the Pax7 + p3b (Figures 6J,P; Moreno et al., 2012). However, in the turtle, but not in *Xenopus* (Figures 6B,C), within the Nkx2.1 expressing region (Figure 6O) a portion that is Isl1-/Otp- (Figure 6K, asterisk in K’) can be distinguished between the Isl1 + Tub and the Otp + and Nkx2.2 + Ma region (Figures 6K,L). It has been discussed (see above) that this region could correspond to the ventral tuberal portion proposed in mammals (Puelles et al., 2012a). In addition, the Ma of both reptiles and amphibians shows scattered Pax7 + cells in the subventricular zone late in development that likely originate in the Pax7 expressing population of the adjacent basal plate of P3 (Figures 6D,G–I,O,Q,Q’; Moreno et al., 2012; Domínguez et al., 2014), coincident with the Otp expression observed in the mammillary region (defined by the lack of Isl1 and the expression of Nkx2.1 and Otp; Moreno et al., 2012; Domínguez et al., 2014). This suggests that the longitudinally organization proposed in mammals, and specifically the rostral mammillary band, could also be present in Xenopus and turtle. In this line, in our models Nkx2.2 cells have been observed in the Ma Otp + zone (Figure 5L) and similarly Dlx and GABA expressing cells (Domínguez et al., 2014), likely from the adjacent p3b. In this context, it could also be possible that in mammals some of the Nkx2.2 expressing cells along the alar–basal boundary could reach the periretromammilar region, where Otp is observed in contrast to the perimammillar portion where only Otp is found (see Figure 8.26D in Puelles et al., 2012a).

The mammillary area is also characterized by the expression of genes of the LIM-HD family, whose combinatorial expression pattern led to propose a new regionalization of this territory in mammals (Shimogori et al., 2010). Thus, distinct nuclei were proposed including a supramammillary nucleus Irx5+, a premammillary nucleus expressing Lhx9/Lef1, a mammillary nucleus expressing Lhx1, and a tuberomammillary terminal zone positive for Lhx6, which is continuous with the diagonal band that separates the alar and basal hypothalamic regions (Shimogori et al., 2010). Comparatively, in *Xenopus* Lhx7 is expressed in the alar hypothalamus and continues ventrally into the mammillary territory (Moreno et al., 2004; Domínguez et al., 2013), and this led to propose the existence of a comparable tuberomammillary terminal zone in anurans (Domínguez et al., 2014). The expression of Lhx1 in the mammillary portion of the Xenopus hypothalamus was defined based on the lack of Isl1 in this portion and the Otp expression (Figure 5D; Domínguez et al., 2014). In addition, Lhx1 is also expressed in the mammillary portion of mouse hypothalamus (Bachy et al., 2002; Shimogori et al., 2010). Of note, in their hypothalamic analysis the group of Shimogori et al. (2010) used the Lhx1 expression to define the MM region (mammillary). However, in the current prosomeric interpretation (Puelles et al., 2012a) the expression of Lhx6 is interpreted in the ventral portion of the tuberal hypothalamus (see Figure 8.9 in Puelles et al., 2012a). Independently of its exact anatomical localization, its position in our models suggests, along with the Nkx2.2 and Pax7 expressing cells described before (Figures 5H,L,Q), a comparable longitudinal hypothalamic band that could be comparable to the rostral perimammillar and periretromammilar band (Puelles et al., 2012a).

Several studies in fishes have also reported the differential expression of LIM genes, such as Lhx6 and Lhx1/5, in the basal hypothalamic territory (Osorio et al., 2005; Menuet et al., 2007). Moreover, a recent study has analyzed the mammillary organization proposed by Shimogori et al. (2010) in zebrafish, finding different domains based on the differential expression of Lef1, Lhx6, Irx5 and Foxb1 (Wolf and Ryu, 2013). Thus, the presence of Otp in the Nkx2.1 positive region of the mammillary band was reported to be playing a crucial role in the establishment of different mammillary domains and is involved in the specification of posterior hypothalamic neurons regulating the expression of Fezf2 and Foxb1.2 in the putative mammillary region (Wolf and Ryu, 2013).

The lack of Shh expression in RMa has also been reported in the chicken, where the Shh becomes downregulated in the tuberomammillary primordium, but not in the RMa, at a specific point during development (Martí et al., 1995; Shimamura et al., 1995; Crossley et al., 2001; Patten et al., 2003; Manning et al., 2006), what seems to confer the hypothalamic fate to these cells (Manning et al., 2006). In the mammillary territory of mammals and birds, two rostro-caudal portions have been described on the basis of the differential expression of Nkx2.1 and Shh. Thus, there is a Nkx2.1+/Shh-region that is also positive for Otp (Bardet et al., 2008; Morales-Delgado et al., 2011), and a Nkx2.1-/Shh+ RMa region (García-Calero et al., 2008; Morales-Delgado et al., 2011). Attending to the Shh/Nkx2.1 expression pattern in the mammillary territory, it represents an exception within the prosencephalon, being the only forebrain area where Shh and Nkx2.1 are not expressed in parallel because Shh expression becomes secondarily downregulated at some point in the development (Shimamura et al., 1995; Crossley et al., 2001; Manning et al., 2006).

Thus, regarding the situation in fishes, some studies have described the presence of Shh in the basal hypothalamus, although so far there are no data about the specific location of Shh expression within this basal hypothalamic territory. It has been reported the presence of two hedgehog genes, expressed in a Sonic Hh-like pattern, in the basal hypothalamus of lamprey (Osorio et al., 2005; Kano et al., 2010). In addition, expression of Shh has been reported in the basal hypothalamus of cavefish and zebrafish (Menuet et al., 2007; Wolf and Ryu, 2013), where its expression seems to be limited in the tuberal territory, although no specific distinction of tuberal and mammillary regions was described.

In terms of chemical specification, the amphibian and reptilian mammillary region is characterized by the presence of a rich catecholaminergic cell population (Smeets et al., 1987; Smeets and González, 2000; Moreno et al., 2012; Domínguez et al., 2014) that co-expresses Nkx2.1 (Figures 6E,G,M,O) and Otp (Figures 6F,G,N,O), in line with previous studies in other vertebrates and suggesting a conserved role of both transcription factors in the specification of the dopaminergic phenotype during the anamnio-amniotic transition (Kawano et al., 2003; Del Giacco et al., 2006; Blechman et al., 2007; Ryu et al., 2007; Löhr et al., 2009).

Regarding the nuclear specification, controversy exists regarding the origin of the different neuronal groups and several data support the contribution of diencephalic areas to the mammillary territory. In mammals, the retromammillary area was considered a caudoventral hypothalamic specification located between the diencephalic tegmentum (in P3; see for review Puelles et al., 2012a), giving rise to the subthalamic nucleus. In birds, recent fate map studies have described that the basal plate of P3 generates the retromammillary tegmentum and the subthalamic nucleus (Garcia-Lopez et al., 2009). In reptiles and anurans, Pax7 expressing cells likely originated in P3 colonize the mammillary region (Moreno et al., 2012; Bandín et al., 2013; Domínguez et al., 2014), suggesting a diencephalic contribution to the formation of the hypothalamic mammillary territory. Moreover, the subthalamic nucleus in mammals was identified by the expression of Pax7 (Stoykova and Gruss, 1994; see mouse developmental Allen Brain Atlas), thus the Pax7 positive cells found dispersed in the mammilary territory in *Xenopus* could suggest the existence of a forerunner of the subthalamic nucleus in anurans (Domínguez et al., 2014).

## Concluding remarks

The organization of the brain undergoes evolutionary/adaptative changes during the anamnio-amniotic transition. The evolutionary leap from amphibians to reptiles involves relevant adaptation changes to conquer a new environment that have clear consequences on brain organization. However, it seems that during the transition from aquatic to terrestrial life the hypothalamus has maintained a major general pattern of organization, but with subtle differences that could be related to the new requirements for adaptation to the new environment. These variations in hypothalamic organization/regionalization highlighted in the present comparative genoarchitectonic analysis appear to have occurred gradually during the anamnio-amniotic transition starting with amphibians, which are the first tetrapods that arose, being anamniotes (Table 1; Figure 7). Considering the data gathered on the organization of the hypothalamus, it seems that there is a mostly common general pattern shared by all vertebrates that includes the following main features: (1) it belongs to the secondary prosencephalon and is topologically rostral to the diencephalon; and (2) it is formed by alar and basal regions that show genoarchitectonic patterns during development that are generally conserved across vertebrates, especially in the basal territories.

In the evolutionary context (Table 1; Figure 7), our results in amphibians and reptiles add information to the known features of the hypothalamic organization in birds and mammals and point out to some main features shared by all tetrapods: (1) each alar (SPV, SC) and basal (Tub, M) territory is also subdivided rostrocaudally into two different domains based on molecular criteria; (2) the expression of Nkx2.1 that characterizes the entire SC region in fishes starts to be restricted in amphibians and is gradually reduced through mammals where the SC virtually lacks expression of this transcription factor, what could be related to the gradual pallial and thalamic expansion that take place in the amniotes. In addition, there are some features in the organization of the hypothalamus that seem to have emerged with the amniotes (see Figure 7): (1) the existence of the preoptohypothalamic boundary observed in amniotes starts in reptiles; (2) also in reptiles, as in birds and mammals, Pax6 is expressed in the SPV, whereas such expression is not observed in anamniotes. These facts highlight the relevance of the studies involving species of amphibians and reptiles for elaborating a complete evolutionary story of the hypothalamus.

**Figure 8:**
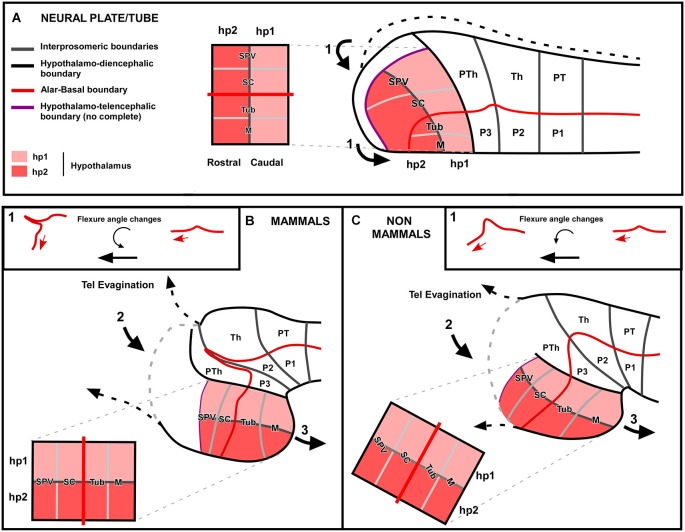
**Schematic comparison of the different forces during ontogeny that lead to the different hypothalamic anatomy**. In this hypothetic scheme the situation between mammals and non-mammals (mainly based on our results in the development of the amphibian hypothalamus) are represented. Three main forces are supposed to act in a sequential manner and differently in each vertebrate group. The first force (1) to act is the flexure of the neural tube **(A)**. In mammals, the longitunal axis bends almost 90° forming a sharp flexure and the rostral tube is moved to a “ventral” position **(B)**, whereas in non-mammals this angle seems to be less pronounced **(C)**. Then, a second morphological force acts over this longitudinal axis that is already partially bent, which is produced by the telencephalic evagination (2). In the case of mammals this second force acts equally on the caudal (hp1) and rostral (hp2) hypothalamic domains, so its main effect would be the flattening of the hypothalamic territory. However, in the case of non-mammals, the strength caused by the telencephalic evagination would be mainly pushing the rostral (hp2) hypothalamic domain, which helps to turn more “ventrally” the hypothalamus. Finally, a third force is the hypothalamic evagination (3). In mammals this third strength is contributing to the elongation of the hypothalamic territory and, in the case of non-mammals this force is also contributing to pronounced hypothalamic modification.

Comparative studies of the hypothalamus across vertebrates encompass many difficulties because the different degree of topographical modification of its parts, due to diverse forces during development that lead to the final different anatomy in each group (Figures 7, 8). The forces involved in the hypothalamic final conformation might be of different nature. If we consider the situation in mammals, in a “non-disturbed” neural tube at the level of the prosencephalon (Figure 8A) the alar hypothalamus is in the most rostral portion along with the telencephalic prospective territories, which will give rise to the telencephalic vesicles and the telencephalon impar during development. The neural tube suffers a second morphological strength given the flexure of the neural tube, which is maximum at the level of the diencephalic basal plate, thus at the boundary with the hypothalamic basal region. In addition, those rostral regions of the brain are under the direct morphological strength that produces the evagination of the telencephalic vesicles. Specially in mammals, the pallium is enormously expanded dramatically increasing in size and literally pushing the adjacent regions, like the alar hypothalamus. Therefore, in mammals due to the drastic expansion of the pallium, together with the strong flexure of the brain that bends the longitudinal axis almost 90°, the hypothalamus acquires a “ventral” position (Figure 8B). In the case of non-mammalian vertebrates, and specially in anamniotes, these developmental changes due to morphological pressures are, in general, less significant (Figure 8C). Telencephalic development is less massive and the cephalic flexure less pronounced, varying in the different vertebrates. However, in spite of the different topography of the hypothalamus, studies such as ours reveal that comparable subdivisions are contained in the hypothalamus of each group. Therefore, the main final conclusion of the comparative analysis of the region of the hypothalamus in vertebrates is probably the high degree of conservation of this region in evolution, as expected given its functional importance in the animal survival. Developmental forces during the ontogeny of each vertebrate group would be responsible for the different topographical arrangement of the hypothalamic regions, which otherwise are similarly specified by gene expression patterns throughout vertebrates.

## Author contributions

All authors had full access to all the data in the study and take responsibility for the integrity of the data and the accuracy of the data analysis. This review is based on previous studies in which the three authors were involved (Moreno et al., 2012; Domínguez et al., 2013, 2014).

## Conflict of interest statement

The authors declare that the research was conducted in the absence of any commercial or financial relationships that could be construed as a potential conflict of interest.

## Abbreviations

BH: basal hypothalamus
CeA: central amygdala
CPa: central portion of the paraventricular area
CT: caudal tuberal region
Dg: diagonal domain
DPa: dorsal portion of the paraventricular area
EPTh: prethalamic eminence
hp1: hypothalamic prosomeric domain 1
hp2: hypothalamic prosomeric domain 2
Hyp: hypophysis
M: mammillary region
Ma: mammillary area proper
MeA: medial amygdala
oc: optic chiasm
P1: prosomere 1
P2: prosomere 2
P3: prosomere 3
P3b: basal plate of P3
Pa: paraventricular nucleus
Pal: pallidum
PM: perimammillary area
PO: preoptic region
POC: preoptocommissural area
POH: preoptohypothalamic boundary
PPa: peduncular domain of Pa
PSPa: peduncular domain of SPa
PT: pretectum
PTh: prethalamus
PR: perimammillary area
PRM: periretromammillary area
RM: retromammillary area
RMa: retromammillary region
RT: rostral tuberal region
RTu: retrotuberal region (peduncular)
RtuD: retrotuberal dorsal domain
RTul: retrotuberal lateral domain
RTuV: retrotuberal ventral domain
SC: suprachiasmatic region
SCc: caudal suprachiasmatic region
SCr: rostral suprachiasmatic region
Spa: subparaventricular area
SP: subpallium
SPV: supraoptoparaventricular region
SPVc: caudal supraoptoparaventricular region
SPVr: rostral supraoptoparaventricular region
Str: striatum
VPa: ventral portion of the paraventricular area
Th: thalamus
TPa: terminal domain of Pa
TPaC: central portion of TPa
TPaD: dorsal portion of TPa
TPaV: ventral portion of TPa
TSPa: terminal domain of SPa
Tu: tuberal region (terminal)
TuD: dorsal tuberal domain
Tul: lateral tuberal domain
TuV: ventral tuberal domain
Tub: tuberal region.

## References

[B1] Abellán A. Medina L. (2009). Subdivisions and derivatives of the chicken subpallium based on expression of LIM and other regulatory genes and markers of neuron subpopulations during development. J. Comp. Neurol. 515, 465–501. 10.1002/cne.22083 19459222

[B2] Abellán A. Vernier B. Rétaux S. Medina L. (2010). Similarities and differences in the forebrain expression of Lhx1 and Lhx5 between chicken and mouse: insights for understanding telencephalic development and evolution. J. Comp. Neurol. 518, 3512–3528. 10.1002/cne.22410 20589911

[B3] Acampora D. Postiglione M. P. Avantaggiato V. Di Bonito M. Vaccarino F. M. Michaud J.. (1999). Progressive impairment of developing neuroendocrine cell lineages in the hypothalamus of mice lacking the Orthopedia gene. Genes Dev. 13, 2787–2800. 10.1101/gad.13.21.2787 10557207 PMC317121

[B4] Alunni A. Blin M. Deschet K. Bourrat F. Vernier P. Rétaux S. (2004). Cloning and developmental expression patterns of Dlx2, Lhx7 and Lhx9 in the medaka fish (Oryzias latipes). Mech. Dev. 121, 977–983. 10.1016/j.mod.2004.03.023 15210202

[B5] Bachy I. Berthon J. Rétaux S. (2002). Defining pallial and subpallial divisions in the developing *Xenopus* forebrain. Mech. Dev. 117, 163–172. 10.1016/s0925-4773(02)00199-5 12204256

[B6] Bandín S. Morona R. Moreno N. González A. (2013). Regional expression of Pax7 in the brain of *Xenopus laevis* during embryonic and larval development. Front. Neuroanat. 7:48. 10.3389/fnana.2013.00048 24399938 PMC3871710

[B7] Bardet S. M. Cobos I. Puelles E. Martínez-De-La-Torre M. Puelles L. (2006). Chicken lateral septal organ and other circumventricular organs form in a striatal subdomain abutting the molecular striatopallidal border. J. Comp. Neurol. 499, 745–767. 10.1002/cne.21121 17048229

[B8] Bardet S. M. Ferrán J. L. Sánchez-Arrones L. Puelles L. (2010). Ontogenetic expression of sonic hedgehog in the chicken subpallium. Front. Neuroanat. 4:28. 10.3389/fnana.2010.00028 20700498 PMC2917215

[B9] Bardet S. M. Martínez-de-la-Torre M. Northcutt R. G. Rubenstein J. L. Puelles L. (2008). Conserved pattern of OTP-positive cells in the paraventricular nucleus and other hypothalamic sites of tetrapods. Brain Res. Bull. 75, 231–235. 10.1016/j.brainresbull.2007.10.037 18331876

[B10] Blechman J. Borodovsky N. Eisenberg M. Nabel-Rosen H. Grimm J. Levkowitz G. (2007). Specification of hypothalamic neurons by dual regulation of the homeodomain protein Orthopedia. Development 134, 4417–4426. 10.1242/dev.011262 18003738

[B11] Bourguignon C. Li J. Papalopulu N. (1998). XBF-1, a winged helix transcription factor with dual activity, has a role in positioning neurogenesis in *Xenopus* competent ectoderm. Development 125, 4889–4900. 9811573 10.1242/dev.125.24.4889

[B12] Briscoe J. Ericson J. (1999). The specification of neuronal identity by graded Sonic Hedgehog signalling. Semin. Cell Dev. Biol. 10, 353–362. 10.1006/scdb.1999.0295 10441550

[B13] Brox A. Puelles L. Ferreiro B. Medina L. (2003). Expression of the genes GAD67 and Distal-less-4 in the forebrain of *Xenopus laevis* confirms a common pattern in tetrapods. J. Comp. Neurol. 461, 370–393. 10.1002/cne.10688 12746875

[B14] Bruce L. (2008). “Evolution of the hypothalamus in amniotes. Evolution and Embryological development of forebrain,” in Encyclopedia of Neuroscience, eds Binder M. D. Hirokawa N. Windhorst U. (Berlin Heidelberg: Springer-Verlag), 1363–1367.

[B15] Bruce L. L. Neary T. J. (1995). The limbic system of tetrapods: a comparative analysis of cortical and amygdalar populations. Brain Behav. Evol. 46, 224–234. 10.1159/000113276 8564465

[B16] Bulfone A. Puelles L. Porteus M. H. Frohman M. A. Martin G. R. Rubenstein J. L. (1993). Spatially restricted expression of Dlx-1, Dlx-2 (Tes-1), Gbx-2 and Wnt-3 in the embryonic day 12.5 mouse forebrain defines potential transverse and longitudinal segmental boundaries. J. Neurosci. 13, 3155–3172. 7687285 10.1523/JNEUROSCI.13-07-03155.1993 PMC6576688

[B17] Bupesh M. Abellán A. Medina L. (2011b). Genetic and experimental evidence supports the continuum of the central extended amygdala and a mutiple embryonic origin of its principal neurons. J. Comp. Neurol. 519, 3507–3531. 10.1002/cne.22719 21800302

[B18] Bupesh M. Legáz I. Abellán A. Medina L. (2011a). Multiple telencephalic and extratelencephalic embryonic domains contribute neurons to the medial extended amygdala. J. Comp. Neurol. 519, 1505–1525. 10.1002/cne.22581 21452208

[B19] Butler A. Hodos W. (2005). “The visceral brain: the hypothalamus and autonomic nervous system,” in Comparative Vertebrate Neuroanatomy: Evolution and Adaptation, ed Sons J. W. (New Jersey: Wiley), 445–467.

[B20] Caqueret A. Coumailleau P. Michaud J. L. (2006). Regionalization of the anterior hypothalamus in the chick embryo. Dev. Dyn. 233, 652–658. 10.1002/dvdy.20372 15844192

[B21] Crossley P. H. Martínez S. Ohkubo Y. Rubenstein J. L. (2001). Coordinate expression of Fgf8, Otx2, Bmp4 and Shh in the rostral prosencephalon during development of the telencephalic and optic vesicles. Neuroscience 108, 183–206. 10.1016/s0306-4522(01)00411-0 11734354

[B22] D’Aniello B. Pinelli C. Jadhao A. G. Rastogi R. K. Meyer D. L. (1999). Comparative analysis of FMRFamide-like immunoreactivity in caiman (*Caiman crocodilus*) and turtle (*Trachemys scripta elegans*) brains. Cell Tissue Res. 298, 549–559. 10.1007/s004410050077 10639745

[B23] Davis A. M. Seney M. L. Stallings N. R. Zhao L. Parker K. L. Tobet S. A. (2004). Loss of steroidogenic factor 1 alters cellular topography in the mouse ventromedial nucleus of the hypothalamus. J. Neurobiol. 60, 424–436. 10.1002/neu.20030 15307147

[B24] Del Giacco L. Pistocchi A. Cotelli F. Fortunato A. E. Sordino P. (2008). A peek inside the neurosecretory brain through Orthopedia lenses. Dev. Dyn. 237, 2295–2303. 10.1002/dvdy.21668 18729222

[B25] Del Giacco L. Sordino P. Pistocchi A. Andreakis N. Tarallo R. Di Benedetto B.. (2006). Differential regulation of the zebrafish orthopedia 1 gene during fate determination of diencephalic neurons. BMC Dev. Biol. 6:50. 10.1186/1471-213X-6-50 17074092 PMC1635040

[B26] Diez-Roux G. Banfi S. Sultan M. Geffers L. Anand S. Rozado D.. (2011). A high-resolution anatomical atlas of the transcriptome in the mouse embryo. PLoS Biol. 9:e1000582. 10.1371/journal.pbio.1000582 21267068 PMC3022534

[B27] Domínguez L. González A. Moreno N. (2010). Sonic hedgehog expression during *Xenopus laevis*forebrain development. Brain Res. 1347, 19–32. 10.1016/j.brainres.2010.06.007 20540934

[B28] Domínguez L. González A. Moreno N. (2011). Ontogenetic distribution of the transcription factor nkx2.2 in the developing forebrain of *Xenopus laevis*. Front. Neuroanat. 5:11. 10.3389/fnana.2011.00011 21415915 PMC3049246

[B29] Domínguez L. González A. Moreno N. (2014). Characterization of the hypothalamus of *Xenopus laevis* during development. II. The basal regions. J. Comp. Neurol. 522, 1102–1131. 10.1002/cne.23471 24122702

[B30] Domínguez L. López J. M. González A. (2008). Distribution of thyrotropin-releasing hormone (TRH) immunoreactivity in the brain of urodele amphibians. Brain Behav. Evol. 71, 231–246. 10.1159/000122835 18382103

[B31] Domínguez L. Morona R. González A. Moreno N. (2013). Characterization of the hypothalamus of *Xenopus laevis* during development. I. The alar regions. J. Comp. Neurol. 521, 725–759. 10.1002/cne.23222 22965483

[B32] Eaton J. L. Glasgow E. (2007). Zebrafish orthopedia (otp) is required for isotocin cell development. Dev. Genes Evol. 217, 149–158. 10.1007/s00427-006-0123-2 17180684

[B33] Eaton J. L. Holmqvist B. Glasgow E. (2008). Ontogeny of vasotocin-expressing cells in zebrafish: selective requirement for the transcriptional regulators orthopedia and single-minded 1 in the preoptic area. Dev. Dyn. 237, 995–1005. 10.1002/dvdy.21503 18330923

[B34] Figdor M. C. Stern C. D. (1993). Segmental organization of embryonic diencephalon. Nature 363, 630–634. 10.1038/363630a0 8510755

[B35] Flames N. Pla R. Gelman D. M. Rubenstein J. L. Puelles L. Marín O. (2007). Delineation of multiple subpallial progenitor domains by the combinatorial expression of transcriptional codes. J. Neurosci. 27, 9682–9695. 10.1523/jneurosci.2750-07.2007 17804629 PMC4916652

[B36] García-Calero E. Fernández-Garre P. Martínez S. Puelles L. (2008). Early mammillary pouch specification in the course of prechordal ventralization of the forebrain tegmentum. Dev. Biol. 320, 366–377. 10.1016/j.ydbio.2008.05.545 18597750

[B37] Garcia-Lopez R. Pombero A. Martinez S. (2009). Fate map of the chick embryo neural tube. Dev. Growth Differ. 51, 145–165. 10.1111/j.1440-169x.2009.01096.x 19379273

[B38] Garcia-Lopez R. Vieira C. Echevarria D. Martinez S. (2004). Fate map of the diencephalon and the zona limitans at the 10-somites stage in chick embryos. Dev. Biol. 268, 514–530. 10.1016/j.ydbio.2003.12.038 15063186

[B39] García-Moreno F. Pedraza M. Di Giovannantonio L. G. Di Salvio M. López-Mascaraque L. Simeone A.. (2010). A neuronal migratory pathway crossing from diencephalon to telencephalon populates amygdala nuclei. Nat. Neurosci. 13, 680–689. 10.1038/nn.2556 20495559

[B40] González A. Northcutt R. G. (2009). An immunohistochemical approach to lungfish telencephalic organization. Brain Behav. Evol. 74, 43–55. 10.1159/000229012 19729895

[B41] González A. Smeets W. J. (1991). Comparative analysis of dopamine and tyrosine hydroxylase immunoreactivities in the brain of two amphibians, the anuran *Rana ridibunda* and the urodele *Pleurodeles waltlii*. J. Comp. Neurol. 303, 457–477. 10.1002/cne.903030311 1672535

[B42] González A. Tuinhof R. Smeets W. J. (1993). Distribution of tyrosine hydroxylase and dopamine immunoreactivities in the brain of the South African clawed frog *Xenopus laevis*. Anat. Embryol. (Berl) 187, 193–201. 10.1007/bf00171750 7902028

[B43] Goshu E. Jin H. Lovejoy J. Marion J. F. Michaud J. L. Fan C. M. (2004). Sim2 contributes to neuroendocrine hormone gene expression in the anterior hypothalamus. Mol. Endocrinol. 18, 1251–1262. 10.1210/me.2003-0372 14988428

[B44] Herget U. Wolf A. Wullimann M. F. Ryu S. (2014). Molecular neuroanatomy and chemoarchitecture of the neurosecretory preoptic-hypothalamic area in zebrafish larvae. J. Comp. Neurol. 522, 1542–1564. 10.1002/cne.23480 24127437

[B45] Herrick C. (1910). The morphology of the forebrain in Amphibian and Reptilia. J. Comp. Neurol. 20, 413–547 10.1002/cne.920200502

[B46] Herrick C. (1948). The Brain of the Tiger Salamander, Ambystina Tigrinum. Chicago: The University of Chigaco Press.

[B47] His W. (1893a). Über das frontale Ende des Gehirnrohrs. Arch Anat Entwickelungsges. Anatomische Abteilung des Arch f.Anat u. Physiol 3, 157–171.

[B48] His W. (1893b). Vorschläge zur Eintheilung des Gehirns. Arch Anat Entwickelungsges. Anatomische Abteilung des Arch f.Anat u. Physiol 3, 172–179.

[B49] Hodos W. (2008). “Evolution of the hypothalamus in amniotes,” in Encyclopedia of Neuroscience, eds Binder M. D. Hirokawa N. Windhorst U. (Berlin Heidelberg: Springer-Verlag), 1361–1363.

[B50] Hökfelt T. Fuxe K. Johansson O. Jeffcoate S. White N. (1975). Distribution of thyrotropin-releasing hormone (TRH) in the central nervous system as revealed with immunohistochemistry. Eur. J. Pharmacol. 34, 389–392. 10.1016/0014-2999(75)90269-1 825379

[B51] Hökfelt T. Martensson R. Björklund A. Kleinau S. Goldstein M. (1984). “Distributional maps of tirosine hydroxilase immunoreactive neurons in the rat brain,” in Classical Transmitters in the CNS. I. Handbook of Chemical Neuroanatomy, eds Björklund A. Hökfelt T. (Amsterdam: Elsevier), 277–386.

[B52] Horjales-Araujo E. Hellysaz A. Broberger C. (2014). Lateral hypothalamic thyrotropin-releasing hormone neurons: distribution and relationship to histochemically defined cell populations in the rat. Neuroscience 277, 87–102. 10.1016/j.neuroscience.2014.06.043 24993479

[B53] Kano S. Xiao J. H. Osório J. Ekker M. Hadzhiev Y. Müller F.. (2010). Two lamprey Hedgehog genes share non-coding regulatory sequences and expression patterns with gnathostome Hedgehogs. PLoS One 5:e13332. 10.1371/journal.pone.0013332 20967201 PMC2954159

[B54] Kawano H. Horie M. Honma S. Kawamura K. Takeuchi K. Kimura S. (2003). Aberrant trajectory of ascending dopaminergic pathway in mice lacking Nkx2.1. Exp. Neurol. 182, 103–112. 10.1016/s0014-4886(03)00030-x 12821380

[B55] Kimura S. Hara Y. Pineau T. Fernandez-Salguero P. Fox C. H. Ward J. M.. (1996). The T/ebp null mouse: thyroid-specific enhancer-binding protein is essential for the organogenesis of the thyroid, lung, ventral forebrain and pituitary. Genes Dev. 10, 60–69. 10.1101/gad.10.1.60 8557195

[B56] Kuhlenbeck H. (1973). The Central Nervous System of Vertebrates (Overall Morphologic Pattern, Vol. 3, Part II). Basel: Karger.

[B57] Kurrasch D. M. Cheung C. C. Lee F. Y. Tran P. V. Hata K. Ingraham H. A. (2007). The neonatal ventromedial hypothalamus transcriptome reveals novel markers with spatially distinct patterning. J. Neurosci. 27, 13624–13634. 10.1523/jneurosci.2858-07.2007 18077674 PMC6673626

[B58] Lechan R. M. Wu P. Jackson I. M. (1986). Immunolocalization of the thyrotropin-releasing hormone prohormone in the rat central nervous system. Endocrinology 119, 1210–1216. 10.1210/endo-119-3-1210 3089766

[B59] Löhr H. Ryu S. Driever W. (2009). Zebrafish diencephalic A11-related dopaminergic neurons share a conserved transcriptional network with neuroendocrine cell lineages. Development 136, 1007–1017. 10.1242/dev.033878 19234064

[B60] López J. M. Domínguez L. González A. (2008). Immunohistochemical localization of thyrotropin-releasing hormone in the brain of reptiles. J. Chem. Neuroanat. 36, 251–263. 10.1016/j.jchemneu.2008.06.006 18674611

[B61] Machluf Y. Gutnick A. Levkowitz G. (2011). Development of the zebrafish hypothalamus. Ann. N Y Acad. Sci. 1220, 93–105. 10.1111/j.1749-6632.2010.05945.x 21388407

[B62] Manning L. Ohyama K. Saeger B. Hatano O. Wilson S. A. Logan M.. (2006). Regional morphogenesis in the hypothalamus: a BMP-Tbx2 pathway coordinates fate and proliferation through Shh downregulation. Dev. Cell 11, 873–885. 10.1016/j.devcel.2006.09.021 17141161

[B63] Marín O. Rubenstein J. L. (2001). A long, remarkable journey: tangential migration in the telencephalon. Nat. Rev. Neurosci. 2, 780–790. 10.1038/35097509 11715055

[B64] Markakis E. A. (2002). Development of the neuroendocrine hypothalamus. Front. Neuroendocrinol. 23, 257–291. 10.1016/s0091-3022(02)00003-1 12127306 PMC3242412

[B65] Martí E. Takada R. Bumcrot D. A. Sasaki H. McMahon A. P. (1995). Distribution of Sonic hedgehog peptides in the developing chick and mouse embryo. Development 121, 2537–2547. 7671817 10.1242/dev.121.8.2537

[B66] Martínez-de-la-Torre M. Pombal M. A. Puelles L. (2011). Distal-less-like protein distribution in the larval lamprey forebrain. Neuroscience 178, 270–284. 10.1016/j.neuroscience.2010.12.030 21185911

[B67] Medina L. (2008). “Evolution and embryological development of forebrain,” in Encyclopedia of Neuroscience, eds Binder M. D. Hirokawa N. Windhorst U. (Berlin Heidelberg: Springer-Verlag), 1172–1192.

[B68] Medina L. Bupesh M. Abellán A. (2011). Contribution of genoarchitecture to understanding forebrain evolution and development, with particular emphasis on the amygdala. Brain Behav. Evol. 78, 216–236. 10.1159/000330056 21860224

[B69] Menuet A. Alunni A. Joly J. S. Jeffery W. R. Rétaux S. (2007). Expanded expression of Sonic Hedgehog in Astyanax cavefish: multiple consequences on forebrain development and evolution. Development 134, 845–855. 10.1242/dev.02780 17251267

[B70] Merchenthaler I. Csernus V. Csontos C. Petrusz P. Mess B. (1988). New data on the immunocytochemical localization of thyrotropin-releasing hormone in the rat central nervous system. Am. J. Anat. 181, 359–376. 10.1002/aja.1001810404 3133939

[B71] Michaud J. L. DeRossi C. May N. R. Holdener B. C. Fan C. M. (2000). ARNT2 acts as the dimerization partner of SIM1 for the development of the hypothalamus. Mech. Dev. 90, 253–261. 10.1016/s0925-4773(99)00328-7 10640708

[B72] Michaud J. L. Rosenquist T. May N. R. Fan C. M. (1998). Development of neuroendocrine lineages requires the bHLH-PAS transcription factor SIM1. Genes Dev. 12, 3264–3275. 10.1101/gad.12.20.3264 9784500 PMC317216

[B73] Morales-Delgado N. Castro-Robles B. Ferrán J. L. Martínez-de-la-Torre M. Puelles L. Díaz C. (2014). Regionalized differentiation of CRH, TRH and GHRH peptidergic neurons in the mouse hypothalamus. Brain Struct. Funct. 219, 1083–1111. 10.1007/s00429-013-0554-2 24337236 PMC4013449

[B74] Morales-Delgado N. Merchán P. Bardet S. M. Ferrán J. L. Puelles L. Diaz C. (2011). Topography of Somatostatin gene expression relative to molecular progenitor domains during ontogeny of the mouse hypothalamus. Front. Neuroanat. 5:10. 10.3389/fnana.2011.00010 21441981 PMC3057523

[B75] Moreno N. Bachy I. Rétaux S. González A. (2004). LIM-homeodomain genes as developmental and adult genetic markers of *Xenopus* forebrain functional subdivisions. J. Comp. Neurol. 472, 52–72. 10.1002/cne.20046 15024752

[B76] Moreno N. Domínguez L. Morona R. González A. (2012). Subdivisions of the turtle Pseudemys scripta hypothalamus based on the expression of regulatory genes and neuronal markers. J. Comp. Neurol. 520, 453–478. 10.1002/cne.22762 21935937

[B77] Moreno N. Domínguez L. Rétaux S. González A. (2008b). Islet1 as a marker of subdivisions and cell types in the developing forebrain of *Xenopus*. Neuroscience 154, 1423–1439. 10.1016/j.neuroscience.2008.04.029 18515014

[B78] Moreno N. González A. (2011). The non-evaginated secondary prosencephalon of vertebrates. Front. Neuroanat. 5:12. 10.3389/fnana.2011.00012 21427782 PMC3049325

[B79] Moreno N. Morona R. López J. M. González A. (2010). Subdivisions of the turtle *Pseudemys scripta* subpallium based on the expression of regulatory genes and neuronal markers. J. Comp. Neurol. 518, 4877–4902. 10.1002/cne.22493 21031557

[B80] Moreno N. Rétaux S. González A. (2008a). Spatio-temporal expression of Pax6 in *Xenopus* forebrain. Brain Res. 1239, 92–99. 10.1016/j.brainres.2008.08.052 18786519

[B81] Morona R. González A. (2008). Calbindin-D28k and calretinin expression in the forebrain of anuran and urodele amphibians: further support for newly identified subdivisions. J. Comp. Neurol. 511, 187–220. 10.1002/cne.21832 18781620

[B82] Murakami Y. Ogasawara M. Sugahara F. Hirano S. Satoh N. Kuratani S. (2001). Identification and expression of the lamprey Pax6 gene: evolutionary origin of the segmented brain of vertebrates. Development 128, 3521–3531. 11566857 10.1242/dev.128.18.3521

[B83] Murphy D. B. Wiese S. Burfeind P. Schmundt D. Mattei M. G. Schulz-Schaeffer W.. (1994). Human brain factor 1, a new member of the fork head gene family. Genomics 21, 551–557. 10.1006/geno.1994.1313 7959731

[B84] Nieuwenhuys R. Voogd J. van Huijzen C. (2008). The Human Central Nervous System. Germany: Springer.

[B85] Northcutt R. G. (1970). The Telencephalon of the Western Painted Turtle (*Chrysemys Picta Bellis*). Chicago: University of Illinois Press.

[B86] Ohyama K. Ellis P. Kimura S. Placzek M. (2005). Directed differentiation of neural cells to hypothalamic dopaminergic neurons. Development 132, 5185–5197. 10.1242/dev.02094 16284116

[B87] Osorio J. Mazan S. Rétaux S. (2005). Organisation of the lamprey ( *Lampetra fluviatilis*) embryonic brain: insights from LIM-homeodomain, Pax and hedgehog genes. Dev. Biol. 288, 100–112. 10.1016/j.ydbio.2005.08.042 16289025

[B88] Patten I. Kulesa P. Shen M. M. Fraser S. Placzek M. (2003). Distinct modes of floor plate induction in the chick embryo. Development 130, 4809–4821. 10.1242/dev.00694 12917296

[B89] Price M. Lemaistre M. Pischetola M. Di Lauro R. Duboule D. (1991). A mouse gene related to Distal-less shows a restricted expression in the developing forebrain. Nature 351, 748–751. 10.1038/351748a0 1676488

[B90] Propper C. R. Jones R. E. Lopez K. H. (1992). Distribution of arginine vasotocin in the brain of the lizard Anolis carolinensis. Cell Tissue Res. 267, 391–398. 10.1007/bf00302978 1600566

[B91] Puelles L. (1995). A segmental morphological paradigm for understanding vertebrate forebrains. Brain Behav. Evol. 46, 319–337. 10.1159/000316271 8564469

[B92] Puelles L. (2001). Brain segmentation and forebrain development in amniotes. Brain Res. Bull. 55, 695–710. 10.1016/s0361-9230(01)00588-3 11595354

[B93] Puelles L. Kuwana E. Puelles E. Bulfone A. Shimamura K. Keleher J.. (2000). Pallial and subpallial derivatives in the embryonic chick and mouse telencephalon, traced by the expression of the genes Dlx-2, Emx-1, Nkx-2.1, Pax-6 and Tbr-1. J. Comp. Neurol. 424, 409–438. 10.1002/1096-9861(20000828)424:3<409::aid-cne3>3.0.co;2-7 10906711

[B94] Puelles L. Martínez S. Martínez-de-la-Torre M. Rubenstein J. L. (2004). “Gene maps and related histogenetic domains in the forebrain and midbrain,” in The Rat Nervous System, ed Paxinos G. 3rd Edn. (San Diego: Elsevier), 3–125.

[B95] Puelles L. Martínez de la Torre M. Bardet S. Rubenstein J. L. (2012a). “Hypothalamus,” in The Mouse Nervous System, eds Watson C. Paxinos G. Puelles L. (London: Academic press. Elsevier), 221–313.

[B96] Puelles L. Martínez de la Torre M. Ferrán J. L. Watson C. (2012b). “Diencephalon,” in The Mouse Nervous System, eds Watson C. Paxinos G. Puelles L. (London: Academic press. Elsevier), 313–337.

[B97] Puelles L. Rubenstein J. L. (1993). Expression patterns of homeobox and other putative regulatory genes in the embryonic mouse forebrain suggest a neuromeric organization. Trends Neurosci. 16, 472–479. 10.1016/0166-2236(93)90080-6 7507621

[B98] Puelles L. Rubenstein J. L. (2003). Forebrain gene expression domains and the evolving prosomeric model. Trends Neurosci. 26, 469–476. 10.1016/s0166-2236(03)00234-0 12948657

[B99] Rohr K. B. Barth K. A. Varga Z. M. Wilson S. W. (2001). The nodal pathway acts upstream of hedgehog signaling to specify ventral telencephalic identity. Neuron 29, 341–351. 10.1016/s0896-6273(01)00210-0 11239427

[B100] Roth M. Bonev B. Lindsay J. Lea R. Panagiotaki N. Houart C.. (2010). FoxG1 and TLE2 act cooperatively to regulate ventral telencephalon formation. Development 137, 1553–1562. 10.1242/dev.044909 20356955 PMC2853852

[B101] Rubenstein J. L. Martínez S. Shimamura K. Puelles L. (1994). The embryonic vertebrate forebrain: the prosomeric model. Science 266, 578–580. 10.1126/science.7939711 7939711

[B102] Ryu S. Mahler J. Acampora D. Holzschuh J. Erhardt S. Omodei D.. (2007). Orthopedia homeodomain protein is essential for diencephalic dopaminergic neuron development. Curr. Biol. 17, 873–880. 10.1016/j.cub.2007.04.003 17481897

[B103] Sánchez-Arrones L. Ferrán J. L. Rodríguez-Gallardo L. Puelles L. (2009). Incipient forebrain boundaries traced by differential gene expression and fate mapping in the chick neural plate. Dev. Biol. 335, 43–65. 10.1016/j.ydbio.2009.08.012 19699194

[B104] Sander M. Paydar S. Ericson J. Briscoe J. Berber E. German M.. (2000). Ventral neural patterning by Nkx homeobox genes: Nkx6.1 controls somatic motor neuron and ventral interneuron fates. Genes Dev. 14, 2134–2139. 10.1101/gad.820400 10970877 PMC316892

[B105] Shimamura K. Hartigan D. J. Martínez S. Puelles L. Rubenstein J. L. (1995). Longitudinal organization of the anterior neural plate and neural tube. Development 121, 3923–3933. 8575293 10.1242/dev.121.12.3923

[B106] Shimogori T. Lee D. A. Miranda-Angulo A. Yang Y. Wang H. Jiang L.. (2010). A genomic atlas of mouse hypothalamic development. Nat. Neurosci. 13, 767–775. 10.1038/nn.2545 20436479 PMC4067769

[B107] Smeets W. J. González A. (2000). Catecholamine systems in the brain of vertebrates: new perspectives through a comparative approach. Brain Res. Brain Res. Rev. 33, 308–379. 10.1016/s0165-0173(00)00034-5 11011071

[B108] Smeets W. J. Jonker A. J. Hoogland P. V. (1987). Distribution of dopamine in the forebrain and midbrain of the red-eared turtle, *Pseudemys scripta elegans*, reinvestigated using antibodies against dopamine. Brain Behav. Evol. 30, 121–142. 10.1159/000118642 3664261

[B109] Smeets W. J. Sevensma J. J. Jonker A. J. (1990). Comparative analysis of vasotocin-like immunoreactivity in the brain of the turtle Pseudemys scripta elegans and the snake *Python regius*. Brain Behav. Evol. 35, 65–84. 10.1159/000115857 2191754

[B110] Stoykova A. Gruss P. (1994). Roles of Pax-genes in developing and adult brain as suggested by expression patterns. J. Neurosci. 14, 1395–1412. 8126546 10.1523/JNEUROSCI.14-03-01395.1994 PMC6577564

[B111] Striedter G. F. (1997). The telencephalon of tetrapods in evolution. Brain Behav. Evol. 49, 179–213. 10.1159/000105936 9096908

[B112] Tao W. Lai E. (1992). Telencephalon-restricted expression of BF-1, a new member of the HNF-3/fork head gene family, in the developing rat brain. Neuron 8, 957–966. 10.1016/0896-6273(92)90210-5 1350202

[B113] ten Donkelaar H. J. (1998a). “Anurans,” in The Central Nervous System of Vertebrates, eds Nieuwenhuys R. ten Donkelaar H. J. Nicholson C. (London: Springer), 1151–1314.

[B114] ten Donkelaar H. J. (1998b). “Reptiles,” in The Central Nervous System of Vertebrates, ed Nieuwenhuys R. (London: Springer), 1315–1524.

[B115] Tessmar-Raible K. Raible F. Christodoulou F. Guy K. Rembold M. Hausen H.. (2007). Conserved sensory-neurosecretory cell types in annelid and fish forebrain: insights into hypothalamus evolution. Cell 129, 1389–1400. 10.1016/j.cell.2007.04.041 17604726

[B116] Toresson H. Potter S. S. Campbell K. (2000). Genetic control of dorsal-ventral identity in the telencephalon: opposing roles for Pax6 and Gsh2. Development 127, 4361–4371. 11003836 10.1242/dev.127.20.4361

[B117] Tsuruo Y. Hökfelt T. Visser T. (1987). Thyrotropin releasing hormone (TRH)-immunoreactive cell groups in the rat central nervous system. Exp. Brain Res. 68, 213–217. 10.1007/bf00255248 3121376

[B118] van den Akker W. M. Brox A. Puelles L. Durston A. J. Medina L. (2008). Comparative functional analysis provides evidence for a crucial role for the homeobox gene Nkx2.1/Titf-1 in forebrain evolution. J. Comp. Neurol. 506, 211–223. 10.1002/cne.21542 18022953

[B119] Wang W. Lufkin T. (2000). The murine Otp homeobox gene plays an essential role in the specification of neuronal cell lineages in the developing hypothalamus. Dev. Biol. 227, 432–449. 10.1006/dbio.2000.9902 11071765

[B120] Wolf A. Ryu S. (2013). Specification of posterior hypothalamic neurons requires coordinated activities of Fezf2, Otp, Sim1a and Foxb1.2. Development 140, 1762–1773. 10.1242/dev.085357 23533176

[B121] Yee C. L. Wang Y. Anderson S. Ekker M. Rubenstein J. L. (2009). Arcuate nucleus expression of NKX2.1 and DLX and lineages expressing these transcription factors in neuropeptide Y(+), proopiomelanocortin(+) and tyrosine hydroxylase(+) neurons in neonatal and adult mice. J. Comp. Neurol. 517, 37–50. 10.1002/cne.22132 19711380 PMC3021751

[B122] Zardoya R. Meyer A. (2001a). The evolutionary position of turtles revised. Naturwissenschaften 88, 193–200. 10.1007/s001140100228 11482432

[B123] Zardoya R. Meyer A. (2001b). On the origin of and phylogenetic relationships among living amphibians. Proc. Natl. Acad. Sci. U S A 98, 7380–7383. 10.1073/pnas.111455498 11390961 PMC34677

[B124] Zhao X. F. Suh C. S. Prat C. R. Ellingsen S. Fjose A. (2009). Distinct expression of two foxg1 paralogues in zebrafish. Gene Expr. Patterns 9, 266–272. 10.1016/j.gep.2009.04.001 19379839

